# Blue-Shifting Hydridic Hydrogen Bonds in Complexes of (Me_3_Si)_3_SiH

**DOI:** 10.1021/acs.jpca.5c05765

**Published:** 2025-10-16

**Authors:** Maximilián Lamanec, Vladimír Špirko, Svatopluk Civiš, Pavel Hobza

**Affiliations:** † Institute of Organic Chemistry and Biochemistry of the Czech Academy of Sciences, Flemingovo náměstí 542/2, Prague 6 160 00, Czech Republic; ‡ IT4Innovations, Všb-Technical University of Ostrava, 17. Listopadu 2172/15, Ostrava-Poruba 708 00, Czech Republic; § J. Heyrovský Institute of Physical Chemistry, Czech Academy of Sciences, Dolejškova 2155/3, Prague 8 18200, Czech Republic

## Abstract

Hydridic hydrogen bonds, formed by X–H···Y interactions with negatively charged hydrogen, expand the conventional view of H-bonding beyond elements that are more electronegative than hydrogen. Using a highly polarizable silane donor (Me_3_Si)_3_SiH, we systematically examined various electron acceptors (σ- and π-hole) and observed both red and blue shifts in the X–H stretching frequency. We provide the first experimental evidence of a blue-shifting hydridic bond and report the largest experimental blue shift for any hydrogen-bonded system. Thermodynamic, spectroscopic, and theoretical analyses show that the dispersion energy is crucial for stabilizing these complexes and reproducing their spectral signatures. Notably, the IR band intensity increases for red-shifting bonds and increases or decreases for blue-shifting hydridic bonds, offering a distinct spectroscopic fingerprint. Adiabatic ALMO-EDA calculations indicate that red shifts in hydridic bonds primarily arise from electrostatics and dispersion rather than charge transfer. It can be thus concluded that protonic as well as hydridic hydrogen bonds exhibit similar spectral manifestations, namely, the red or blue shift of the X–H stretching frequency connected with the intensity increase or decrease. These findings broaden hydrogen-bonding paradigms for diverse chemical applications.

## Graphical abstract

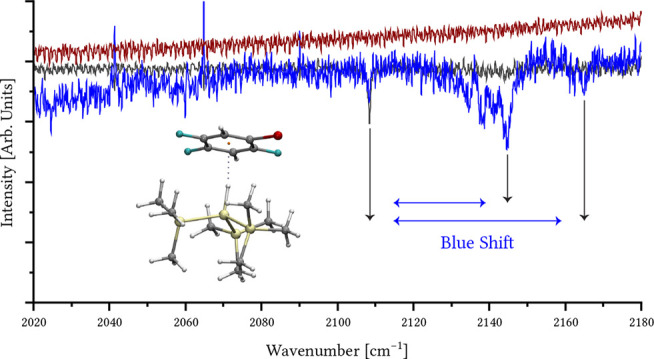

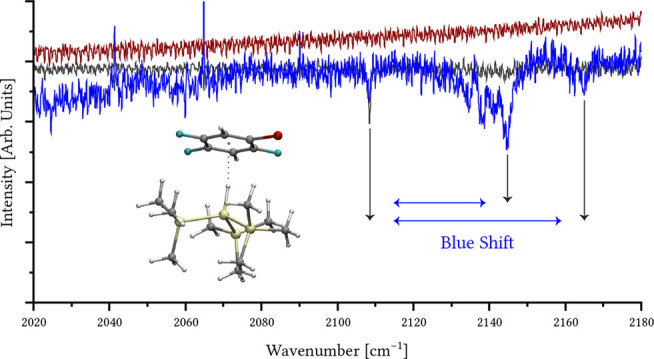

## Introduction

In 2000, we published a paper entitled “Blue-Shifting Hydrogen Bond”, documenting an unconventional hydrogen bond (H-bond) that exhibits a blue shift in the X–H stretching frequency, rather than the classical red shift.^1^ Four years after publishing our review, it was noted that “This review provoked a flurry of publications identifying blue-shifting H-bonds in a number of systems and proposing a variety of explanations.^2^” Initial evidence that the red-shift paradigm could be violated appeared even earlier, in the work of Shraga Pinchas.^3,4^ The first detailed study of a blue-shifting H-bond focused on the chloroform···fluorobenzene complex, where MP2 calculations predicted a blue shift in the C–H stretching frequency—subsequently confirmed by IR spectroscopy in the gas phase.^5^ The largest observed blue shift, 27 cm^–1^, was reported for the fluoroform···acetone-*d*_6_ complex.^6^ Early experimental and computational investigations additionally highlighted another significant departure from red-shifting H-bonds: in certain cases, the intensity of the X–H spectral band diminishes upon H-bond formation.^5,6^ Since then, the term “blue-shifting hydrogen bond” has appeared in more than 1500 publications, and its discovery spurred, among others, the introduction of a previously missing definition for X–H···Y H-bonding. Note that a red shift reflects a decrease in vibrational frequency, whereas a blue shift reflects an increase; these shifts are typically interpreted as evidence of X–H-bond weakening and strengthening, respectively. According to this definition, the involved hydrogen must be bound to an atom X, “where X is more electronegative than H.^7^” The periodic table lists 118 identified elements, yet only 23 of them possess an electronegativity higher than or equal to that of hydrogen.^8^ This restriction confines H-bonding to a limited section of the periodic table. We recognize that these 23 elements—including N, O, F, and C—are fundamental to Earth’s life-supporting cycles. Nonetheless, the chemistry of hydrogen is more diverse. When hydrogen is bound to an element less electronegative than itself, it acquires a negative charge, enabling it to form noncovalent interactions with electrophilic regions, in contrast to the conventional H-bond where hydrogen interacts with nucleophilic regions. Due to this role reversal in the interaction—in which hydrogen becomes partially negative while the H-bond acceptor is partially positive or contains a positive region—this interaction has been termed a hydride bond,^9,10^ an inverse H-bond,^11^ a charge-inverted H-bond,^12–17^ hydridic bond,^18^ or a halogen-hydride bond.^19^ In our previous studies^20–22^ as well as here, we use the term “hydridic H-bond.” We advocate for using the term “hydridic hydrogen bond” for all complexes containing hydridic hydrogen, rather than adopting the IUPAC recommendation to classify them on the basis of the atom acting as the Lewis acid, i.e., as halogen-,^23^ chalcogen-,^24^ or pnictogen bonds.^25^ This choice underscores the parallels with standard (protonic) H-bonds—namely, that hydrogen, the lightest element, is covalently bound to a significantly heavier atom. This characteristic is evident not only in IR spectra but, to some extent, also in NMR fingerprints of H-bonding. The Et_3_GeH···ICF_3_ complex that we recently described provides a representative case study.^22^ This species can be viewed either as a hydridic Ge–H···I hydrogen-bonded complex or as a H···I–C halogen-bonded complex. Formation of the halogen bond produces only a trivial red shift (≈3 cm^–1^) in the I–C stretching frequency and leaves its intensity essentially unchanged. By contrast, when the interaction is analyzed as a “H-bond”, a pronounced red shift (≈48 cm^–1^) in the Ge–H stretching frequency is observed together with a marked increase in band intensity. The question thus arises whether it is better to use for this and similar complexes the recommended name of the halogen bond where experimental detection of the respective properties would be difficult.

Although a blue shift might initially seem counterintuitive to the established understanding of H-bonding, multiple explanations have been proposed. These include models based on Natural Bond Orbitals (NBO),^1^ electron-density analysis,^26^ rehybridization,^27^ dispersion,^28^ and a more recent approach using Adiabatic Absolutely Localized Molecular Orbitals Energy Decomposition Analysis (ALMO-EDA).^29^ The latter suggests that when charge transfer is restricted during geometry optimization, H-bonds consistently exhibit a blue shift. As a result, charge transfer has been identified as the primary factor driving the red shift and bond elongation in classical H-bonds. Changes of intensity of the X–H spectral band are connected with changes of the X–H dipole moment upon formation of the X–H···Y H-bond. While the red-shifting protonic H-bonding is mostly connected with an intensity increase,^28^ the situation with blue-shifting protonic H-bonding characterized by an increase as well as a of intensity is less clear.

In our recent papers, we demonstrated that hydridic H-bonded complexes, specifically Me_3_SiH with BrCN and ICF_3_ ^20^ as well as Et_3_GeH with ICF_3_,^22^ exhibit a red shift in their X–H stretching frequencies and an increase in the intensity of the respective spectral band upon complexation. Several previous theoretical studies have predicted blue-shifting behavior for hydridic H-bonds, see, e.g., paper by Jablonski,^18^ who wrote *“··· that the stretching vibration frequency of a hydridic R-H bond can also reveal either the red or the blue shift*.” However, up to now, there has been no systematic theoretical and experimental confirmation of this phenomenon. In order to investigate the potential for such shifts, we selected in the current study the (Me_3_Si)_3_SiH as an electron donor due to its high polarizability and systematically examined its interactions with a broad range of electron acceptors: σ-hole acceptors (BrCN, BrSO_2_CF_3_, ICF_3_, ICN, P­(CN)_3_, and S­(CN)_2_), noble-gas electron vacancies (XeF_4_, Xe­(C_6_F_5_)_2_), and π-hole acceptors (BF_3_, C_6_F_6_, C_6_BrF_3_H_2_, C_6_ClF_5_, C_6_(CN)_3_H_3_, C_6_(CN)_6_, C_3_N_3_(CF_3_)_3_, COF_2_, and NO_2_F). For three electron acceptors (C_6_F_6_, C_6_BrF_3_H_2_, and C_6_ClF_5_), IR spectra in the Ar matrix are provided. Our combined experimental and theoretical investigations reveal both red and blue shifts in the Si–H stretching frequency of (Me_3_Si)_3_SiH upon complexation, culminating in the largest blue shift of an X–H stretching frequency yet observed via the low-temperature IR spectroscopy among both types of H-bonds.

In addition to frequency shifts, the calculations predict changes in the intensity of the Si–H stretching bands: red-shifted hydridic H-bonds exhibit increased intensity, whereas blue-shifted hydridic H-bonds may either intensify or decrease. This study marks a further step in our ongoing comparison of protonic and hydridic H-bonding. We deliberately selected a title closely mirroring one used 25 years ago to underscore the striking similarities between these two classes of H-bonds.^1^

## Methods

### IR Experiments

In our low-temperature IR experiments, we employed two main approaches. The first approach, termed the solid-phase complex (SPC) method, involves the direct spectral measurement of a supersonically expanded mixture of reaction intermediates deposited on a cold substrate. The second approach utilizes noble-gas matrix isolation.

A detailed description of these low-temperature experiments appears in ref 30. Briefly, gas mixtures were deposited onto a cooled KBr substrate (18–70 K) inside a Leybold cryostat chamber. We monitored the relative concentrations of products by integrating the absorption intensities of the selected IR bands. In the SPC method, the intermediates were supersonically expanded into a high vacuum (∼10^–6^ Torr) and deposited onto the substrate at 18 K (the lowest attainable temperature). Spectra were recorded using a Bruker Vertex spectrometer equipped with KBr optics, an HgCdTe detector, and a KBr beam splitter. Optical interference filters refined the broad 700–5000 cm^–1^ range, and the KBr entrance window of the spectrometer was employed. The unapodized spectral resolution was 0.06 cm^–1^, and each sample required 1000–1500 coadded scans to achieve an acceptable signal-to-noise ratio. We calibrated observed wavenumbers against rotation-vibration lines of H_2_O in the spectra. Matrix isolation, frequently used to stabilize ions, radicals, and other transient species, employs a similar principle. A mixture of reaction intermediates diluted in argon (molar ratio of 1:500) was pulsed onto a 20 K KBr substrate, and the spectra were recorded with the same Bruker Vertex spectrometer setup.

The experimental resolution of hydrogen bond intensities is often nontrivial owing to the properties of the individual components that comprise the molecular complex. One critical factor affecting both complex formation and its identification is the partial pressure of the gaseous components. In the case of (Me_3_Si)_3_SiH···fluorobenzene complexes, (Me_3_Si)_3_SiH poses a particular challenge because of its relatively low partial pressure (1 Torr). During sample preparation, it is essential to carefully mix each component both with itself and subsequently with the inert gas (Ar). Our results show that the stoichiometric ratio is paramount for successful complex formation. Another key factor is achieving as high a total pressure as possible prior to supersonic expansion; in our setup, using a glass vacuum apparatus for mixing, we reached nearly atmospheric pressure.

A further consideration is the thickness of the deposited layer on a KBr substrate inside of the cryostat. For all three complexes in Figure 1, sensitivity was maximized by increasing the total spectrometer scans to 1200 and applying a relatively thick matrix layer. Only then could we detect the spectral shift of the Si–H transition. Experimentally, we confirmed that the X–H vibration in blue-shifting complexes is relatively weak in absorption, consistent with quantum mechanical predictions, indicating a decrease in band intensity.

**Figure 1:**
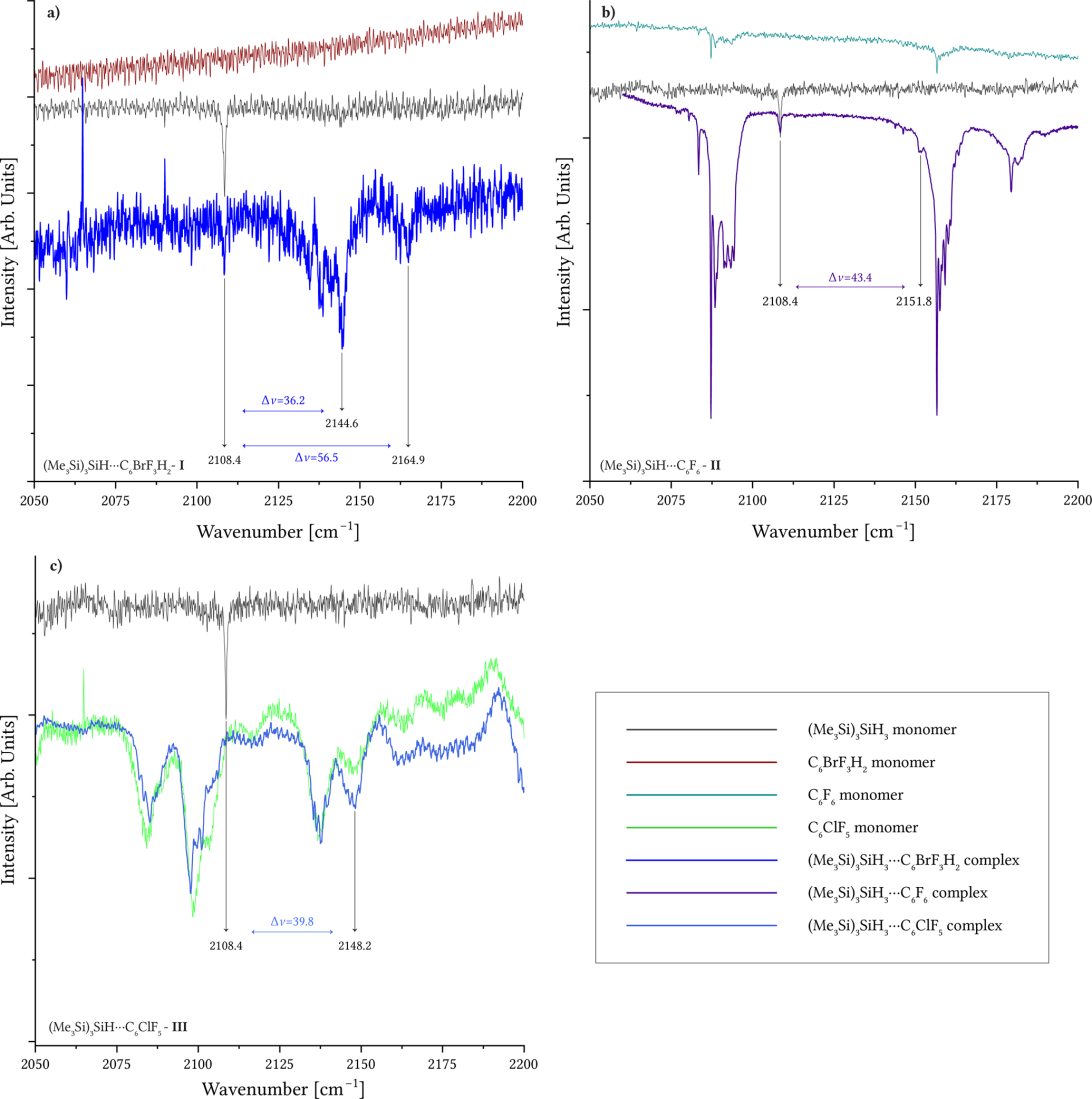
IR spectra in an argon matrix compare each (Me_3_Si)_3_SiH complex with its respective monomers. IR spectra in an Ar matrix of the investigated complexes. Panel **a** shows spectra of the (Me_3_Si)_3_SiH···C_6_BrF_3_H_2_ complex (dark blue), along with the monomer (Me_3_Si)_3_SiH (gray) and the monomer C_6_BrF_3_H_2_ (red). Panel **b** shows spectra of the (Me_3_Si)_3_SiH···C_6_F_6_ complex (violet), along with the monomer (Me_3_Si)_3_SiH (gray) and the monomer C_6_F_6_ (cyan). Panel **c** shows spectra of the (Me_3_Si)_3_SiH···C_6_ClF_5_ complex (green), along with the monomer (Me_3_Si)_3_SiH (gray) and the monomer C_6_ClF_5_ (light blue).

Although we could not precisely quantify these intensity changes, we compared the Si–H vibration and its intensity to precursor bands in the spectra. Even when the ratios and combinations of the components were varied, those precursor bands remained present in the spectra of blue-shifting complexes, with a high-band-intensity ratio relative to the Si–H complex absorption. This observation indicates that all blue-shifting (Me_3_Si)_3_SiH···X complexes display relatively weak absorption, in line with theoretical predictions.

In contrast, for red-shifting complexes, we reinvestigated the IR spectra of Me_3_SiH···ICF_3_ in the solid phase (see details below, cf. Figure 3). In this system, we directly measured the spectra of a solid-ice film on the KBr substrate, enabling us to assess the complex’s stability over a temperature range and determine its decomposition temperature (140 K) with high precision.

All chemicals were purchased from commercial sources (Sigma-Aldrich spol. s r. o. and Apollo Scientific Ltd.) and used without further purification.

### Calculations

Geometry optimizations for all subsystems and complexes were performed using the MP2 and HF methods with the RIJCOSX^31^ approximation and the cc-pwCVTZ^32^ basis set (cc-pwCVTZ-PP^33^ for Br, I, and Xe). Additionally, the ωB97M-V^34^ functional and the PBE0 ^35^ functional with D4 ^36^ dispersion correction and the RIJCOSX approximation, both with the def2-TZVPPD^37^ basis set, were employed. We chose MP2 as widely used for H-bond complexes also in our previous publications,^20,22^ ωB97M-V to its widely accurate describing of various interactions,^38^ and PBE0 as widely used cheap DFT functional with reasonably good results. Harmonic frequencies and intensities were calculated at the same levels of theory as the geometry optimizations using the rigid-rotor harmonic oscillator approximation. NBO analysis was performed using NBO5 ^39^ implemented in Q-Chem 6.2.^40^ NCI^41^ analysis was performed using Multiwfn 3.8 software.^42^ For the calculation of partial charges, we employed the Atomic Dipole Moment Corrected Hirshfeld (ADCH) population method,^43^ which has been reported to provide the best correlation with experimental data.^44^ Partial charges were calculated using Multiwfn 3.8, based on wave functions obtained from Q-Chem at the ωB97M-V/def2-TZVPPD level.

Because the complexes presented in this work include a larger number of atoms than the complexes reported previously,^21,22^ we first conducted a benchmark (see Table S1) by performing SAPT^45^ (2 + 3) calculations on MP2-optimized complexes of Me_3_SiH with ICF_3_, BrCN, and C_6_F_6_, as well as on related C_3v_-constrained structures. We then employed more economic SAPT2+ in subsequent calculations for (Me_3_Si)_3_SiH complexes, as it showed good agreement with SAPT­(2 + 3). Although SAPT2+ slightly overestimates electrostatic, induction, and repulsion contributions and underestimates dispersion by ∼1 kcal·mol^–1^, it still maintains a clear dominance of the dispersion contribution over the electrostatic contribution.

For the adiabatic ALMO-EDA,^46^ we employed the ωB97M-V functional with the def2-TZVPPD basis set. However, due to the high computational cost, it was not feasible to apply this approach to the (Me_3_Si)_3_SiH complexes with C_6_(CN)_3_H_3_, C_6_(CN)_6_, C_3_N_3_(CF_3_)_3_, or Xe­(C_6_F_5_)_2_. MP2 and PBE0 calculations were performed using the ORCA 6.0.0 software package.^47^ ALMO-EDA and ωB97M-V calculations were carried out with a Q-Chem 6.2. SAPT calculations were performed at various levels of theory and with multiple basis sets using the PSI4 1.9 software package.^48^

All visualizations were done using VMD 1.9.3.^49^

### Anharmonic Calculations

Just as in our previous publications,^20,50,51^ the evaluation of the sought vibrational Si–H stretching frequencies and corresponding vibrational wave functions relies on the deep adiabatic separability of the high-frequency Si–H mode from the rest of the vibrational degrees of freedom of the probed complexes, on the assumption of a purely linear dependence of the coordinates of the optimized atomic positions of these complexes on the stretching Si–H distortion and on the structural similarity of these complexes. Under these assumptions, the sought quantities are obtainable by solving a one-dimensional vibrational Schrödinger equation with the Si–H minimum energy pass stretching potentials and a constant reduced mass that can be used as a single scaling parameter. The reduced mass actually used in this study was determined by the requirement to reproduce the experimental Si–H frequency shift in the (Me_3_Si)_3_SiH···C_6_F_6_ complex. As can be seen in Table 1, the thus-obtained reduced mass provides Si–H spectral shifts of the remaining probed complexes in good agreement both with their experimental values and the values obtained within the framework of the harmonic approximation. Importantly, the same harmony is also faced in the case of the relative intensities *I*_rel_ of the probed Si–H fundamental vibrational bands. The “anharmonic” values of these intensities were evaluated using the following relation:

$$I_{\mathrm{rel}}=\frac{{\left(\langle \psi_{0}\left(r\right)|d_{\mathrm{complex}}|\psi_{1}\left(r\right)\rangle \right)}^{2}}{{\left(\langle \psi_{0}\left(r\right)|d_{\mathrm{monomer}}|\psi_{1}\left(r\right)\rangle \right)}^{2}}$$

**Table 1:** Experimental and Theoretical Data for Complexes of (Me_3_Si)_3_SiH with Substituted Benzenes^a^

MP2							
I (Me_3_Si)_3_SiH···C_6_BrF_3_H_2_							
Δν_EXP_	Structure	Δν_CALC_	*I*_C_/*I*_M_ ^b^	Δ*E* ^T^	Δ*G* (20 K)	Δ*r*	*r*(H···π)
36.2	**Ia**	58.0(49.5)	0.6(0.6)	–8.54	–7.62	–0.005	2.403
	**Ib**	52.9(50.5)	0.6(0.5)	–8.82	–6.76	–0.004	2.427
56.5	**Ic**	71.8(58.0)	0.5(0.7)	–8.55	–7.70	–0.006	2.350
	**Id**	68.1(61.3)	0.5(0.8)	–8.58	–6.23	–0.006	2.358
**II (Me_3_Si)_3_SiH···C_6_F_6_**							
43.4	**II**	78.1(43.5)	0.7(0.8)	–9.34	–8.35	–0.006	2.302
**III (Me_3_Si)_3_SiH···C_6_ClF_5_**							
39.8	**IIIa**	72.6(56.7)	0.7(0.7)	–9.84	–8.81	–0.006	2.315
	**IIIb**	70.6(51.2)	0.7(0.8)	–9.81	–8.73	–0.006	2.322

ωB97M-V							
I (Me_3_Si)_3_SiH···C_6_BrF_3_H_2_							
Δν_EXP_	Structure	Δν_CALC_	*I*_C_/*I*_M_ ^b^	Δ*E* ^T^	Δ*G* (20 K)	Δ*r*	*r*(H···π)
36.2	**Ia**	70.7	0.8	–6.44	–6.06	–0.005	2.490
	**Ib**	65.7	0.8	–6.37	–6.03	–0.005	2.499
56.5	**Ic**	63.8	0.8	–6.32	–5.85	–0.005	2.515
	**Id**	69.5	0.8	–6.50	–6.53	–0.005	2.514
**II (Me_3_Si)_3_SiH···C_6_F_6_**							
43.3	**II**	42.1	1.1	–6.67	–6.16	–0.001	2.555
**III (Me_3_Si)_3_SiH···C_6_ClF_5_**							
39.8	**IIIa**	45.4	1.1	–7.13	–6.56	–0.001	2.555
	**IIIb**	61.2	1.0	–6.96	–6.46	–0.003	2.466

a Experimentally observed IR shift of the Si–H stretching frequency (Δν_EXP_, in cm^–1^), calculated harmonic shift of the Si–H stretching frequency (Δν_CALC_, in cm^–1^) (anharmonic values in parentheses), harmonic intensity ratio of the Si–H stretching band (*I*_C_/*I*_M_) (anharmonic values in parentheses), total interaction energy (Δ*E* ^T^, in kcal·mol^–1^), Gibbs free energy at 20 K (Δ*G*, in kcal·mol^–1^), calculated change in the Si–H bond length upon complexation (Δ*r*, in Å), and the distance between the hydridic hydrogen and the substituted benzene center of Ring (*r*(H···π),in Å). All calculations were performed at the MP2/cc-pwCVTZ level (cc-pwCVTZ-PP for Br and I) and at the ωB97M-V/def2-TZVPPD level of theory.

b Ratio between the intensity of the Si–H band in the complex (*I*_C_) and that of the monomer (*I*_M_).

where *d*_monomer_ and *d*_complex_ are the appropriate “energy minimum path” theoretical dipole moment functions, and ψ_0_(*r*) and ψ_1_(*r*) are the wave functions of the probed vibrational ground and first excited states. Theoretical dipole moment functions were obtained by calculating the dipole moment for both the monomer and the complex, where the Si–H bond was either elongated (up to +1.5 Å from the equilibrium geometry, in steps of 0.05 Å) or shortened (down to −0.7 Å from the equilibrium geometry, in steps of 0.05 Å). Pointwise theoretical dipole moment functions were smoothed with cubic splines. The calculated “energy minimum path” potential energy *V*(Si–H)) and electric dipole moment μ­(Si–H) functions and the corresponding wave functions of the ground and first excited vibrational states (Ψ_0_ and Ψ_1_) of the Si–H fragment of the probed complexes strongly coincide with their monomeric counterparts shown in Figure S1.

## Results and Discussion

### IR Spectra in Ar Matrix

Figure 1 presents the IR spectra at 20 K of (Me_3_Si)_3_SiH, each acceptor molecule, and their corresponding complexes in a single arrangement: Panel a compares pure (Me_3_Si)_3_SiH (gray), pure C_6_BrF_3_H_2_ (red), and the (Me_3_Si)_3_SiH···C_6_BrF_3_H_2_ complex (dark blue), revealing 36 and 57 cm^–1^ blue shifts in the Si–H stretch upon complexation; Panel b shows pure (Me_3_Si)_3_SiH (gray), pure C_6_F_6_ (cyan), and the (Me_3_Si)_3_SiH···C_6_F_6_ complex (violet), displaying a shift of 43 cm^–1^; and Panel c illustrates pure (Me_3_Si)_3_SiH (gray), pure C_6_ClF_5_ (green), and the (Me_3_Si)_3_SiH···C_6_ClF_5_ complex (light blue), which exhibits a 40 cm^–1^ shift in the Si–H stretching frequency upon complex formation. The peaks in Panel b (2087, 2156, 2179 cm^–1^) and in Panel c (2084, 2098, 2137, 2161 cm^–1^) are combination bands that arise exclusively from halogenated benzene monomer fundamental modes and were assigned earlier.^52,53^

### Structural, Energetic, and Vibrational Characteristics

Figures 2 and S2–S4 show the optimized structures for the studied complexes, while Tables 1 and 2 list their computed properties for those investigated experimentally and theoretically, respectively. We focus on MP2, selected for its agreement with experiments and distinguishing between minima of complex (**I**) and ωB97M-V calculations for the (Me_3_Si)_3_SiH···C_6_BrF_3_H_2_ (**I**), (Me_3_Si)_3_SiH···C_6_F_6_ (**II**), and (Me_3_Si)_3_SiH···C_6_ClF_5_ (**III**) complexes, all characterized via low-temperature IR spectroscopy.

**Figure 2:**
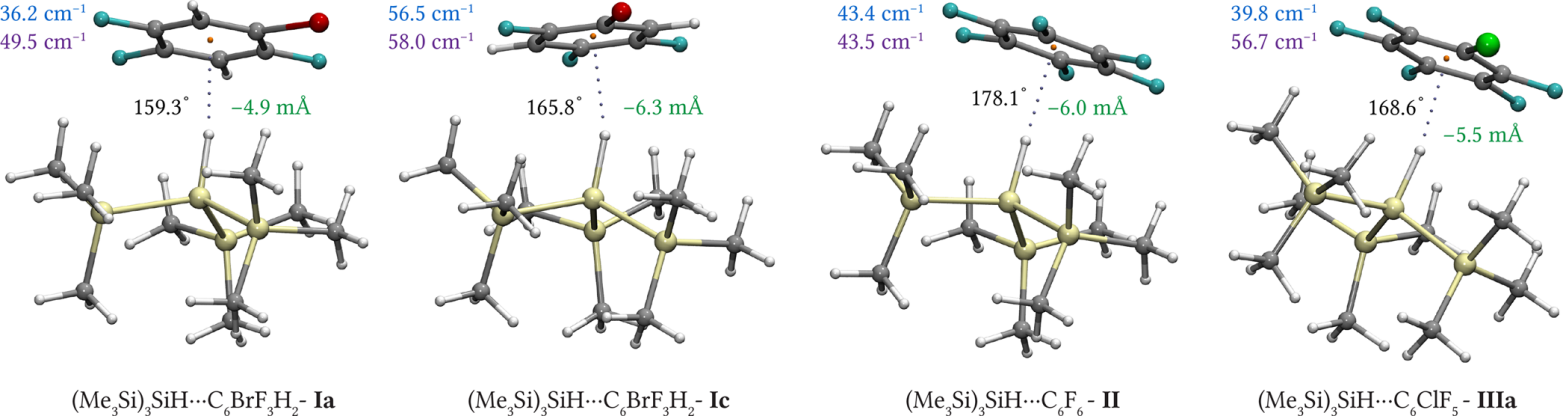
Optimized geometries of blue-shifting (Me_3_Si)_3_SiH complexes with substituted benzenes. MP2/cc-pwCVTZ (cc-pwCVTZ-PP for Br)-optimized structures of four blue-shifting complexes—(Me_3_Si)_3_SiH···C_6_BrF_3_H_2_ (**Ia** and **Ic**), (Me_3_Si)_3_SiH···C_6_F_6_ (**II**), and (Me_3_Si)_3_SiH···C_6_ClF_5_ (**IIIa**). Here, green numbers denote the Si–H bond length change upon complexation, black numbers represent the angle formed by Si, the hydridic H, and the center of mass of benzene derivatives (orange dot), blue numbers denote the experimentally measured blue shift of the Si–H stretching frequency upon complexation, and purple numbers denote the calculated anharmonic value of the Si–H shift on the MP2 level (C: gray, Si: light yellow, H: white, Br: dark red, Cl: green, and F: cyan).

**Table 2:** Total Interaction Energy (Δ*E* ^T^), Calculated Harmonic Shift of the Si–H Stretching Frequency (Δν_CALC_, in cm^–1^), Calculated Harmonic Intensity Ratio of the Si–H Stretching Band (*I*_C_/*I*_M_), Total Interaction Energy (Δ*E* ^T^, in kcal·mol^–1^), and Calculated Change in the Si–H Bond Length upon Complexation (Δ*r*, in Å) of Hydridic H-Bond Complexes (Me_3_Si)_3_SiH···Y’^a^

	MP2				ωB97M-V			
Y‘	Δ*E* ^T^	Δν_CALC_	*I*_C_/*I*_M_ ^b^	Δ*r*	Δ*E* ^T^	Δν_CALC_	*I*_C_/*I*_M_ ^b^	Δ*r*
BF_3_	–4.09	–0.5	2.2	0.002	–4.56	–11.5	2.3	0.005
BrCN	–4.47	–29.2	2.5	0.005	–3.99	–15.9	2.4	0.005
BrSO_2_CF_3_	–5.48	–29.3	2.3	0.004	–4.88	–30.8	2.2	0.004
ICF_3_	–4.81	–28.9	2.6	0.008	–4.89	–23.3	2.6	0.005
ICN	–5.86	–48.2	3.5	0.009	–5.81	–28.4	3.7	0.009
P(CN)_3_	–9.18	–52.7	6.2	0.004	–7.80	–43.7	6.0	0.009
S(CN)_2_	–6.32	–21.1	2.8	–0.006	–5.22	–8.9	3.0	0.004
C_6_(CN)_3_H_3_	–9.98	77.5	0.9	–0.001	–7.68	74.4	1.1	–0.004
C_6_(CN)_6_	–14.84	58.0	1.7	–0.001	–11.46	49.2	1.9	0.001
C_3_N_3_(CF_3_)_3_	–11.91	46.0	1.8	–0.001	–9.65	54.6	1.8	0
COF_2_	–4.20	8.7	1.6	0.001	–3.78	6.7	1.6	0.001
NO_2_F	–4.76	17.0	1.2	–0.001	–0.18	11.5	1.3	0
XeF_4_	–7.18	13.6	2.0	0	–5.00	1.1	2.0	0.003
Xe(C_6_F_5_)_2_	–13.49	36.5	1.2	–0.003	–10.15	29.1	1.3	–0.001

a All calculations were performed at the MP2/cc-pwCVTZ Level (cc-pwCVTZ-PP for Br and I) and at the ωB97M-V/def2-TZVPPD level of theory.

b Ratio between the intensity of the Si–H band in the complex (*I*_C_) and that of the monomer (*I*_M_).

Despite sharing the same electron donor, complexes **I**, **II**, and **III** differ slightly in their structures and properties. As depicted in Figure 2 and Table 1, each exhibits a shortened Si–H bond upon complexation, commensurate with the observed blue shifts of 36, 57, 43, and 40 cm^–1^, respectively. These values align well with those of anharmonic MP2 calculations. The binding free energies exceed 5 kcal·mol^–1^ at 20 K, confirming stability under experimental conditions.

For complex **I** ((Me_3_Si)_3_SiH···C_6_BrF_3_H_2_), four local minima (**Ia**, **Ib**, **Ic**, **Id**) were identified (Figure S4), each showing an Si–H···π-hole contact but differing in the orientation of C_6_BrF_3_H_2_ above (Me_3_Si)_3_SiH. Their binding free energies vary by 1.5 kcal·mol^–1^. In agreement with the experiment, we identified two minima (**Ia** and **Ib**) with respective shifts of 50 and 51 cm^–1^ (anharmonic) and 58 and 53 cm^–1^ (harmonic) align well with the first observed experimental shifted band at 36 cm^–1^. The other two minima for complex **I** (**Ic** and **Id**) have close shifts of the Si–H stretching frequencies 58 and 61 (anharmonic) and 68 and 61 (harmonic) align well with the measured 57 cm^–1^ shift. By contrast, the high symmetry of the monomers C_6_F_6_ and (Me_3_Si)_3_SiH (D_6_h and C_3v_) in complex **II** yields a single minimum, producing one narrow IR band in agreement with the MP2 harmonic value (78 cm^–1^). Complex **III** ((Me_3_Si)_3_SiH···C_6_ClF_5_) has two nearly isoenergetic minima (**IIIa** and **IIIb**), both contributing to a single band at 40 cm^–1^, resulting in 71 and 73 cm^–1^ (harmonic) and 51 and 57 cm^–1^ (anharmonic). ωB97M-V calculations confirm these findings, yielding minor differences in Δν­(Si–H) across the complexes. Only the MP2 anharmonic treatment reproduces the largest experimental blue shift observed for structure **Id**. By contrast, harmonic calculations at the MP2 level erroneously assign the largest blue shift to structure **II**, in clear disagreement with the measurements.

Remarkably, these experimentally verified blue shifts are not only the first reported for hydridic H-bonded complexes but also the largest for any H-bonded system measured at low temperature. Previously, the highest documented blue shift was 27 cm^–1^ in complexes such as fluoroform···acetone*-d*_6_ ^6^ and fluoroform···*p*-cyanophenol,^54^ whereas complex **I** surpasses this by more than a factor of 2. Although the experimental setup does not permit direct measurement of Si–H band intensity changes upon complexation, Table 1’s computational data suggest decreases or negligible intensity increases, consistent with analogous findings in blue-shifting protonic H-bonds. It should be stressed that only experimentally verified blue shifts are considered.^6^

Table 2 presents results on hydridic H-bonded complexes of (Me_3_Si)_3_SiH with 14 different electron acceptors determined at the MP2 and ωB97M-V levels. We employed both σ-hole and π-hole electron acceptors. The depiction of σ-holes and π-holes in the monomers, as well as the negative values of the electrostatic potential (ESP) on the electron donors, is shown in Figures S5 and S6. Complexes of BF_3_, BrCN, BrSO_2_CF_3_, ICF_3_, ICN, P­(CN)_3_, and S­(CN)_2_ show a red shift along with a significant elongation of the Si–H bond, while the remaining complexes show a blue shift accompanied by a shortened Si–H bond. All complexes exhibit an increase of intensity which is significantly larger for the red-shifted H-bonds. These results were also confirmed at the PBE0-D4/def2-TZVPPD level of theory (Table S2).

Several additional minima were located for the experimentally studied complexes (Figure S7). Their key properties are compiled in Table S3 (MP2) and Table S4 (ωB97M-V). Owing to the proximity of the silyl groups and the substituted benzenes, we conclude that most of these structures are stabilized predominantly by dispersion interactions. Only in the complex (Me_3_Si)_3_SiH···C_6_BrF_3_H_2_ have we also identified a Si–H···Br contact, representing a Si–H···σ-hole interaction. Dispersion-bound complexes are less stable than those featuring a Si–H···π-hole contact and have a negligible effect on the Si–H stretching frequency. Although such species could coexist—because the substituted benzene engages a different region of the silane—their minimal influence on the Si–H band makes them experimentally invisible. The alternative conformer (Me_3_Si)_3_SiH···C_6_BrF_3_H_2_ competes with the Si–H···π-hole structure but is higher in energy by more than 2 kcal·mol^–1^, so its population is expected to be very low. This conclusion is consistent with the experiment: the red shift anticipated for a Si–H···Br interaction (−14.3 cm^–1^) is absent from the spectrum in Figure 1.

### Role of Dispersion in Blue-Shifting Hydridic Bond

Using the Symmetry-Adapted Perturbation Theory (SAPT) which provides reliable values of individual energy components, like electrostatic, induction, and dispersion, we pointed out^21,22^ the dominant role of dispersion interactions in hydridic H-bonded complexes. This was explained by the presence of a partial negative charge at hydridic hydrogen, making this hydrogen more polarizable compared to protonic hydrogen.^21^ An alternative explanation was made by Roberts and Mao^55^ who explained the dominant role of dispersion energy by steric bulk within the silane hydrogen donor using the ALMO-EDA method. Table S5 shows that also for all (Me_3_Si)_3_SiH complexes, the dispersion energy exceeds both electrostatic and induction contributions and can be nearly twice as large as the electrostatic term in certain blue-shifting complexes. For the smaller, less polarizable Me_3_SiH, the corresponding ratio is lower. The dispersion energy of the (Me_3_Si)_3_SiH···C_6_F_6_ complex is more than twice that of the analogous Me_3_SiH···C_6_F_6_ complex, a difference that stems not only from the nine methyl groups but also from the three highly polarizable Si–Si bonds. As noted above, dispersion forces drive the two subsystems into closer contact—even at the price of an energetically unfavorable shortening of the X–H bond—which manifests experimentally as a blue shift.

It has been noted that HF theory is often sufficient to reproduce the blue shift in standard H-bonded complexes;^56^ however, hydridic H-bonds appear to rely more heavily on dispersion (i.e., Coulombic correlation). Because HF lacks Coulombic correlation energy,^57^ it struggles to capture these significant dispersion effects. To explore the impact of the dispersion energy on both interaction energy and Si–H vibrational shifts, Table S6 compares HF and MP2 results for several hydridic H-bonded systems. While HF can reproduce the blue shift observed in standard H-bonds, it fails to do so in hydridic ones, where dispersion is critical. In red-shifting hydridic complexes, HF underestimates the magnitude of the red shift relative to MP2 and, in nearly all cases, fails to predict the blue shift that MP2 predicts.

### Origin of Blue Shift

The pronounced blue shift observed—and accurately predicted—for structure **Ic** cannot be explained by conventional charge transfer arguments. Shifts in X–H stretching frequencies are usually ascribed to electron donation either into the σ* antibonding orbital of the electron acceptor (protonic H-bonds) or from the σ bonding orbital of the electron donor (hydridic H-bonds). Our Natural Bond Orbital (NBO) analysis (Tables S7 and S8) shows that complex formation induces a meaningful change just for red-shifting complexes. Occupation in the σ bonding orbital is lowering, thus resulting in elongation of the Si–H bond and a red shift. It corresponds with similar findings in our previous work.^22^ For blue-shifting complexes, there are no meaningful changes neither in the σ-bonding orbitals of the hydride donor nor in the σ* antibonding orbital of the electron acceptor—evidence that charge transfer between these orbitals is negligible for blue-shifting complexes. Consequently, the Si–H bond contraction and its accompanying blue shift must arise from a different mechanism.

As illustrated in Figure 2, every blue-shifted complex adopts a stacked geometry in which the plane defined by the six peripheral methyl hydrogens and the central Si–H hydrogen lies parallel to the substituted benzene ring. The methyl hydrogens carry a positive partial charge (+0.10 e), whereas the Si–H hydrogen bears a negative partial charge (−0.03 e). For comparison, the hydridic hydrogen in Me_3_SiH carries a partial charge of −0.08 e, and in SiH_4_, a partial charge of −0.04 e. A weak electrostatic attraction between this negatively charged hydrogen and the substituted benzene π-hole, reinforced by dispersion interactions—amplified by the six methyl groups and the three highly polarizable Si–Si bonds of (Me_3_Si)_3_SiH—draws the two subunits into unusually close contact. This enforced proximity creates a ″repulsion wall” and shortens the Si–H bond, producing the observed blue shift in the Si–H stretching frequency. An NCI plot highlighting these noncovalent contacts is provided in Figure S8. According to the NCI plot shown in Figure S9, there are distinct contributions from weak attractive interactions (green regions, −0.005 to −0.015 au) and weak repulsive interactions (red regions, 0.003 to 0.015 au).

### Origin of Red Shift

Similarly, as in the work of Roberts and Mao,^55^ we employed the adiabatic ALMO-EDA method at the ωB97M-V/def2-TZVPPD level, performing geometry optimizations on both polarized (POL) and frozen (FRZ) surfaces designed to remove the effects of polarization (POL) and to remove both charge transfer and polarization (FRZ). On the FRZ surface, only electrostatics and Pauli repulsion are included. Dispersion contributions are accounted for through the nonlocal correlation described by the ωB97M-V functional.

Using adiabatic ALMO-EDA, we examined first H-bonded complexes of chloroform with pyridine, ammonia, and water—systems that exhibit red shifts upon full optimization—and systems with benzene, fluorobenzene, and sulfane, which show blue shifts. For the red-shifting complexes, the POL surface optimization induced a shortened C–H bond, leading to a blue shift upon complexation (see Table S10). Conversely, blue-shifting complexes displayed an even greater blue shift on the POL surface and the C–H bond shortened further after complexation. On the FRZ surface, C–H bonds shortened more dramatically in all complexes, and the blue shift intensified. These findings are consistent with previous reports suggesting that charge transfer is solely responsible for the red shift in H-bonds where fluoroform acts as the electron acceptor.^29^

Turning to hydridic H-bonds, we studied Me_3_SiH complexes with BF_3_, BrCN, C_6_F_6_, C_6_H_3_(CN)_3_, COF_2_, ICF_3_, ICN, NO_2_F, P­(CN)_3_, and S­(CN)_2_, in addition to (Me_3_Si)_3_SiH complexes with BF_3_, BrCN, BrSO_2_CF_3_, ICF_3_, ICN, P­(CN)_3_, S­(CN)_2_, C_6_F_6_, C_5_BrF_5_, COF_2_, NO_2_F, and XeF_4_. Despite the inherently higher computational cost of adiabatic ALMO-EDA at the ωB97M-V level of theory, we observed that Me_3_SiH complexes consistently undergo red shifts on both POL and FRZ surfaces, with concomitant Si–H bond elongation. A similar pattern emerged for red-shifting (Me_3_Si)_3_SiH complexes, as summarized in Table 3: BrCN, BrSO_2_CF_3_, ICF_3_, and ICN complexes exhibited red shifts with Si–H bond elongation on the POL surface, whereas BF_3_ showed a negligible blue shift and P­(CN)_3_ and S­(CN)_2_ produced modest blue shifts (∼10 cm^–1^). For blue-shifting (Me_3_Si)_3_SiH complexes with fluorinated benzenes, the blue shift diminished slightly, paired with Si–H bond elongation, likely reflecting destabilization arising from interactions between the fluorinated aromatic ring and the methyl-group hydrogens of (Me_3_Si)_3_SiH. As stated above, the intermolecular proximity of monomers in blue-shifting complexes is driven by electrostatics and dispersion, which together give rise to the blue shift. This interpretation is supported by the results in Table 3, where the blue-shifting complexes on the FRZ surface maintain their proximity solely due to these two attractive contributions in the FRZ optimization: electrostatics and dispersion. Overall, in contrast to classical H-bonds with fluoroform as the electron acceptor^29^ and with chloroform as the electron acceptor (as described above), adiabatic ALMO-EDA indicates that red shifts in the majority of hydridic H-bonded complexes originate from electrostatic and dispersion interactions.

**Table 3:** Shift in Si–H Stretching Frequency (Δν, in cm^–1^), the Change of Intensity of Si–H Stretching Band (*I*_C_/*I*_M_), the Change in Si–H Bond Length (Δ*r*, in Å) upon Complexation of (Me_3_Si)_3_SiH···Y’ Complexes are Compared to the Fully Optimized (Me_3_Si)_3_SiH^a^

	FULL			POL			FRZ		
Y’	Δν	*I*_C_/*I*_M_ ^b^	Δ*r*	Δν	*I*_C_/*I*_M_ ^b^	Δ*r*	Δν	*I*_C_/*I*_M_ ^b^	Δ*r*
BF_3_	–11.5	2.3	0.0054	0.2	1.6	0.0061	–3.9	0.6	0.0022
BrCN	–15.9	2.4	0.0046	–2.5	1.8	0.0025	–3.5	1.0	0.0019
BrSO_2_CF_3_	–30.8	2.2	0.0043	–5.6	1.5	0.0016	–1.5	1.0	0.0008
ICF_3_	–23.3	2.6	0.0048	–8.5	1.8	0.0025	1.4	1.0	0.0001
ICN	–28.4	3.7	0.0086	–2.8	2.3	0.0043	–8.6	1.0	0.0034
P(CN)_3_	–43.7	6.0	0.0088	11.2	2.9	0.0027	7.9	0.9	0.0013
S(CN)_2_	–8.9	3.0	0.0042	9.4	2.0	0.0019	3.0	0.9	0.0005
C_6_F_6_	42.1	1.1	–0.0011	39.6	1.3	–0.0009	35.1	0.8	–0.0009
C_6_ClF_5_	45.4	1.1	–0.0011	47.6	1.2	–0.0013	36.0	0.8	–0.0009
C_6_BrF_3_H_2_ ^c^	62.5	0.8	–0.0051	61.0	1.0	–0.0044	55.4	0.8	–0.0026
COF_2_	6.7	1.6	0.0014	11.4	1.5	0.0006	8.5	0.9	0.0002
NO_2_F	11.5	1.3	0.0004	16.7	1.3	0.0000	12.5	0.9	–0.0001
XeF_4_	1.1	2.0	0.0030	4.0	1.8	0.0024	1.6	0.9	0.0018

a Results are presented for full optimization (FULL) at the ωB97M-V/def2-TZVPPD level, as well as adiabatic ALMO-EDA optimizations on polarized (POL) and frozen (FRZ) surfaces.

b Ratio between the intensity of the Si–H band in the complex (*I*_C_) and that of the monomer (*I*_M_).

c Due to convergence problem, data for (Me_3_Si)_3_SiH···C_6_BrF_3_H_2_ complex were calculated in def2-TZVPP basis set.

### Intensity Changes upon H-Bond Formation

Changes in the intensity of the X–H stretching band, alongside shifts in its frequency, represent a key spectroscopic signature of H-bond formation. In the present study, we were unable to experimentally detect changes in the Si–H band intensity of (Me_3_Si)_3_SiH upon hydridic H-bond formation, primarily due to practical limitations. However, computational methods readily capture such intensity variations. In our previous work,^22^ for instance, we investigated 13 hydridic H-bonded complexes that displayed red shifts and found a consistent increase in the Si–H band intensity—an increase that, however, was not confirmed experimentally.

Although precise quantification of intensity changes in blue-shifting complexes proved challenging, we compared the Si–H vibrational band to the precursor bands in the spectra. These reference bands persist across various mixing ratios and conditions, implying that the overall absorption by blue-shifting (Me_3_Si)_3_SiH···X complexes is relatively weak—consistent with theoretical predictions. In contrast, for the red-shifting Me_3_SiH···ICF_3_ complex, we obtained spectra in the solid phase (Figure 3), enabling measurement of the complex band alongside the precursor silane band and to precisely determine the decomposition temperature of the studied complex (140 K). The Si–H intensity in the complex exceeded that of the free silane by approximately 7-fold, matching well with the previously calculated 2-fold increase at the MP2/cc-pwCVTZ,^20^ thus lending credibility to the computational approach.

**Figure 3:**
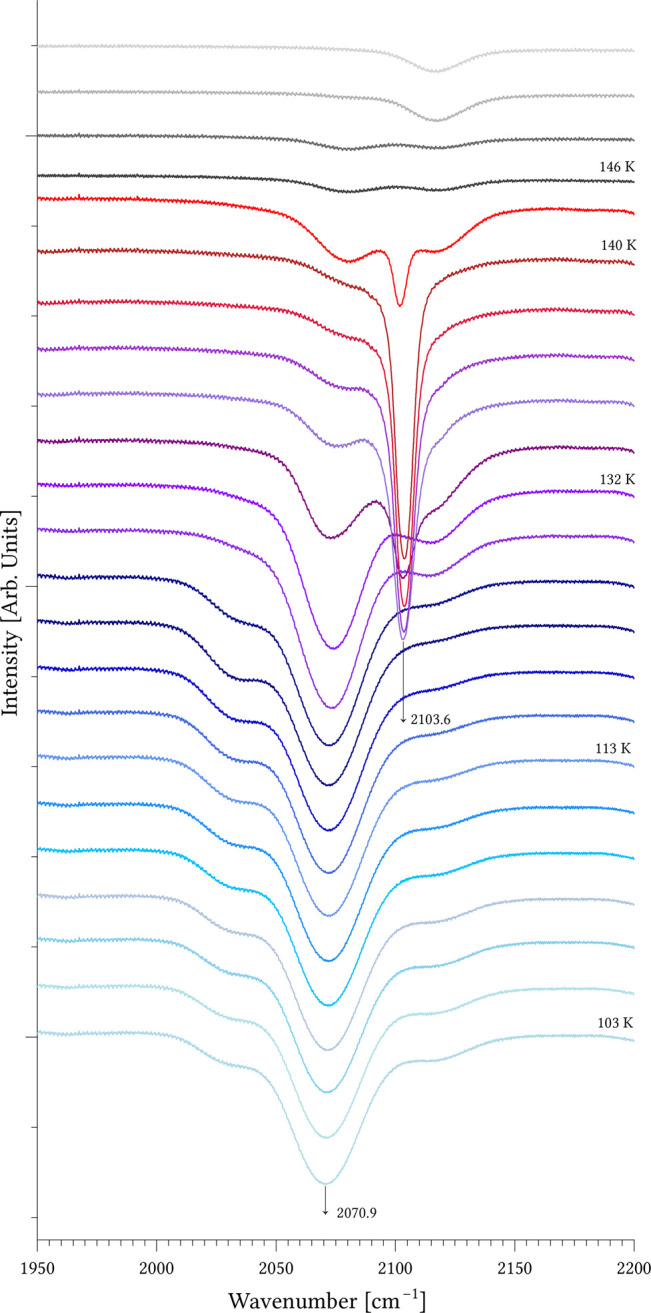
Spectra of Me_3_SiH···ICF_3_ showing the temperature decomposition of the Me_3_SiH···ICF_3_ complex and the intensity change upon the complexation of Me_3_SiH for the Si–H stretching frequency. Thermal decomposition reveals the Si–H stretching frequency of the complex (shades of blue), a mixture of the complex and monomeric Me_3_SiH (shades of violet), and monomeric Me_3_SiH (shades of red). The complex decomposes into its individual components at 140 K. At this temperature, the absorption peak of Me_3_SiH is clearly visible, corresponding precisely to the amount of bound Me_3_SiH in the complex at its decomposition. By integrating the areas of both peaks—Me_3_SiH (140 K) and the complex (103 K)—we obtain an intensity ratio of 1:7, which is consistent with the theoretical increase in intensity of the complex in the case of a red shift.

Complexes **I**, **II**, and **III** (Table 1) deviate notably from other known hydridic H-bonded systems. Surprisingly, we observed a blue shift of the Si–H stretch accompanied by a decrease in band intensity—marking the first documented case of hydridic bonds exhibiting a blue shift and intensity reduction. Although derived from computational evidence, the consistency between theory and experiment seen in red-shifting systems reinforces the reliability of these predictions. Indeed, Table 2 shows that among 17 computationally investigated hydridic complexes, 10 undergo a blue shift, with 1 displaying an intensity decrease according to MP2. In the remaining blue-shifting complexes, the intensity increases are notably smaller than those observed for red-shifting complexes.

Additional insight into IR intensity changes comes from adiabatic ALMO-EDA calculations. Our ωB97M-V results on fully optimized hydridic and protonic complexes reveal consistent intensity increases (see Tables 3, S8 and S9). On the polarized (POL) surface, the intensity remains significantly enhanced, whereas on the frozen (FRZ) surface—where both charge transfer and polarization are excluded—it either remains unchanged or decreases. This outcome suggests that charge transfer and polarization, rather than the nature of the hydrogen (protic vs hydridic) or the direction of the frequency shift (red vs blue), predominantly dictate the enhancement of IR band intensities.

### Hydridic H-Bond or σ-Hole Bond?

All the studied complexes featuring an electron acceptor with a σ-hole can be classified either as σ-hole-bonded (i.e., halogen-, chalcogen-, or pnictogen-bonded), following the recommendation of Arunan et al.,^58^ or as hydridic H-bonded complexes. We favor the latter terminology from a practical standpoint, particularly for IR detection. As shown in Table S11, shifts in X–C or X–S stretching frequencies are very small compared to the more pronounced changes in the Si–H band upon complexation. Likewise, the intensities of these lower-frequency modes remain nearly unchanged, whereas the Si–H stretching band exhibits a significant intensity increase akin to that observed in conventional H-bonds. Consequently, identifying the hydridic H-bond around 2100 cm^–1^ spectroscopically is straightforward and less ambiguous than detecting a σ-hole bond. Furthermore, the X–C or X–S frequencies are localized below 1000 cm^–1^ and these IR measurements are often hindered by limitations in instrumentation, reduced sensitivity, and poor signal-to-noise ratios, compounded by spectral congestion from low-frequency vibrational modes, such as bending, inversion, torsion, and ring puckering.

## Conclusion

In this work, we have demonstrated—both computationally and also, for the first time, experimentally—that hydridic H-bonds can exhibit either red or blue shifts in the X–H stretching frequency. By selecting the highly polarizable (Me_3_Si)_3_SiH as an electron donor (hydride donor) and examining its complexes with a wide range of electron-acceptor (hydride acceptor) molecules, we not only provided the first experimental evidence for blue-shifting hydridic H-bonds but also observed the largest blue shift reported for any H-bonded system thus far. Further, all red-shifted hydridic H-bonded complexes studied computationally have shown an increase of intensity of the Si–H spectral band while a few blue-shifted hydridic H-bonded complexes exhibited a decrease. A decrease was also found for all blue-shifted hydridic H-bonded complexes studied experimentally. The change of the X–H frequency upon formation of the X–H···Y H-bond connected with the change of intensity of the respective spectral band, both being observable, are considered as fingerprints of the formation of H-bond. It can be thus concluded that protonic as well as hydridic H-bonds exhibit similar spectral manifestations, namely the red or blue shift of the X–H stretching frequency connected with the intensity increase or decrease.

Furthermore, our results reveal that strong dispersion interactions typical for hydridic H-bonds (as shown here and in our previous works^21,22^ for more than 60 complexes) are crucial for stabilizing these complexes and producing the observed blue shift, underscoring the need for correlated theoretical methods that correctly account for Coulombic correlation—thereby highlighting why HF theory fails to reproduce the blue shift. Adiabatic ALMO-EDA calculations indicate that red shifts in most hydridic H-bonded complexes are driven by electrostatics and dispersion, with charge transfer providing a significant additional contribution. In most cases, polarization plays only a negligible role, in contrast to charge transfer. Only in two cases—complexes of (Me_3_Si)_3_SiH with P­(CN)_3_ and S­(CN)_2_—are the red shifts driven solely by charge transfer, similar to what we observed in protonic H-bonded complexes with chloroform as the electron acceptor, and as previously reported for fluoroform as the electron acceptor.^29^

To summarize, the results of this study considerably expand our understanding of noncovalent interactions involving negatively polarized (hydridic) hydrogen and provide both experimental benchmarks and robust computational protocols for future explorations of hydridic H-bonded systems.

## Supplementary Material



## Data Availability

Cartesian coordinates for all optimized structures are available at: https://github.com/mlamanec/BlueShiftingHydridicHydrogenBond. All data are available in the main text or the Supporting Information.

## Notes

“Energy minimum path” for anharmonic calculations (Figure S1); Optimized geometries of blue- and red-shifting (Me_3_Si)_3_SiH complexes (Figures S2–S4); molecular electrostatic potential (ESP) maps for electron donors and σ-/π-hole acceptors (Figures S5 and S6); alternative minima with non-Si–H···π contacts (Figure S7); NCI gradient isosurfaces and RDG scatter plots (Figures S8 and S9). Tables S1–S13: SAPT benchmarks (SAPT2+ vs SAPT2 + 3); PBE0-D4 and ωB97M-V energetics; SAPT2+ energy-decomposition on MP2 geometries; HF vs MP2 comparisons; NBO orbital occupancies and second-order perturbation energies (E_2_); adiabatic ALMO-EDA analyses (FULL/POL/FRZ); and computed vibrational frequencies and band intensities (Si–H) across methods (PDF)
