# Supplementary material for: Blue-Shifting Hydridic Hydrogen Bonds in Complexes of (Me3Si)3SiH
Source: J Phys Chem A. 2025 Oct 16;129(50):11512–22. doi: 10.1021/acs.jpca.5c05765 (PMC12720233; doi:10.1021/acs.jpca.5c05765)
Supplement: Supplementary file 1 [file jp5c05765_si_001.pdf]

Blue-Shifting Hydridic Hydrogen Bonds in Complexes of $(Me_3Si)_3SiH$ Supporting information

Maximilián Lamanec,[†,‡] Vladimír Špirko,[†] Svatopluk Civiš,[*,¶] and Pavel Hobza[*,†,‡]

†*Institute of Organic Chemistry and Biochemistry, Czech Academy of Sciences, Flemingovo Náměstí 542/2, 16000 Prague, Czech Republic.*

‡*IT4Innovations, VŠB-Technical University of Ostrava, 17. listopadu 2172/15, 70800 Ostrava-Poruba, Czech Republic*

¶*J. Heyrovský Institute of Physical Chemistry, Czech Academy of Sciences, Dolejškova 2155/3, 18200, Prague 8, Czech Republic*

E-mail: svatopluk.civis@jh-inst.cas.cz; pavel.hobza@uochb.cas.cz

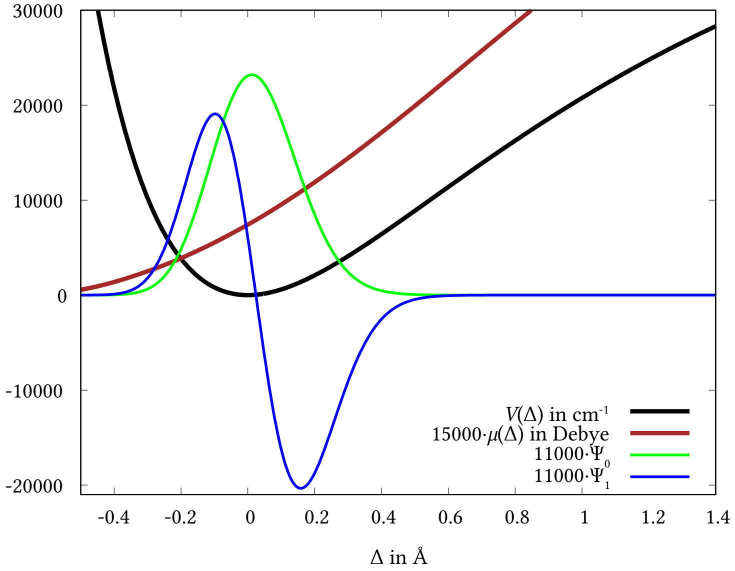

Figure S1: Potential energy function (V), dipole moment function (μ) and vibrational wavefunctions Ψ_0 and Ψ_1 of the ground and first excited vibrational states of $(Me_3Si)_3SiH$.

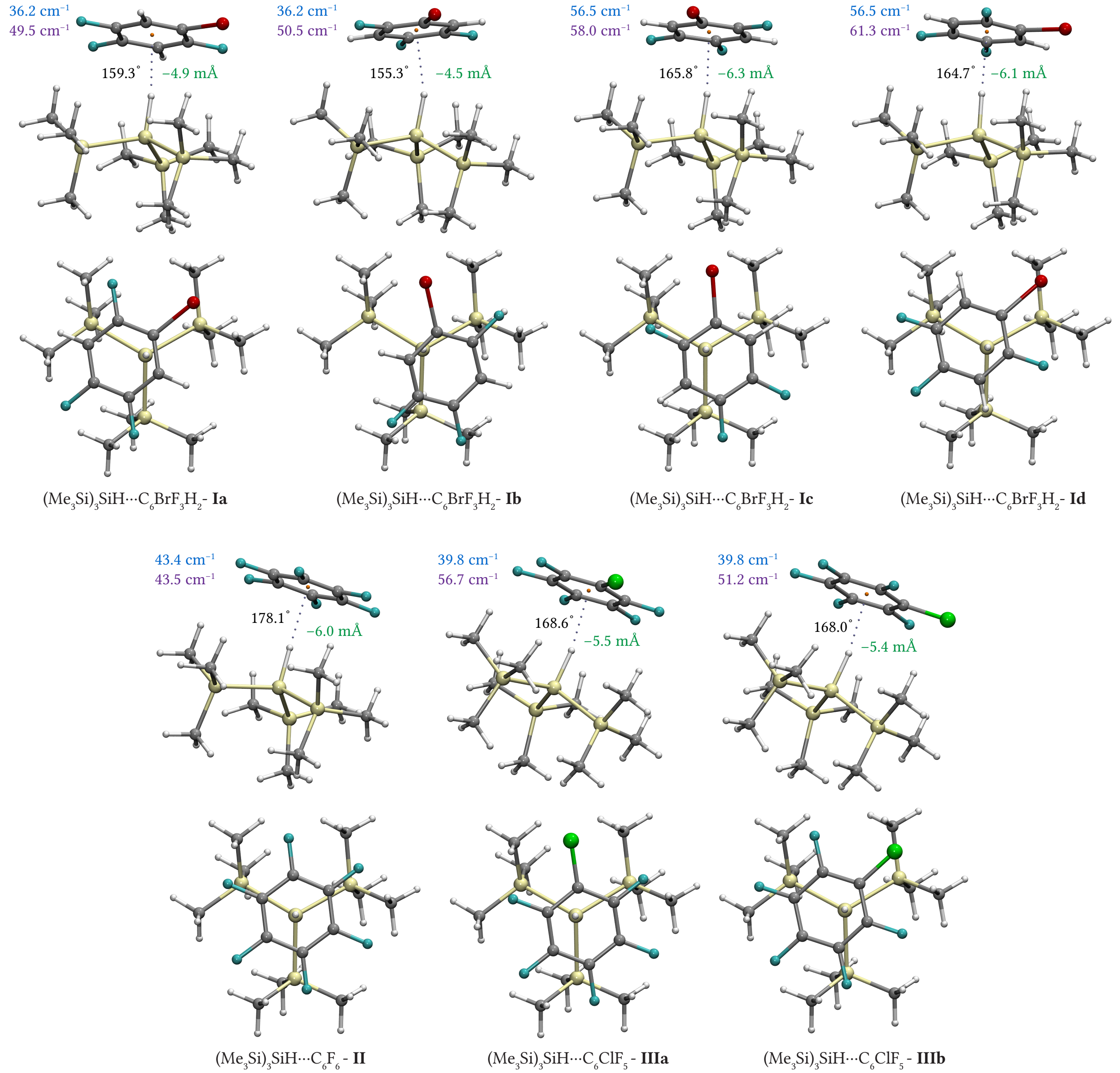

Figure S2: MP2/cc-pwCVTZ (cc-pwCVTZ-PP for Br)-optimized structures of four blue-shifting complexes—$(Me_3Si)_3SiH\cdots C_6BrF_3H_2$ (**Ia**, **Ib**, **Ic** and **Id**), $(Me_3Si)_3SiH\cdots C_6F_6$ (**II**), and $(Me_3Si)_3SiH\cdots C_6ClF_5$ (**IIIa** and **IIIb**). Here, green numbers denote the Si–H bond length change upon complexation, black numbers represent the angle formed by Si, the hydridic H, and the center of mass of benzene derivates (orange dot), blue numbers denote experimentally measured blue shift of Si-H stretching frequency upon complexation and purple numbers denote the calculated anharmonic value of Si-H shift on MP2 level. [C: grey, Si: light yellow, H: white, Br: dark red, Cl: green, and F: cyan.]

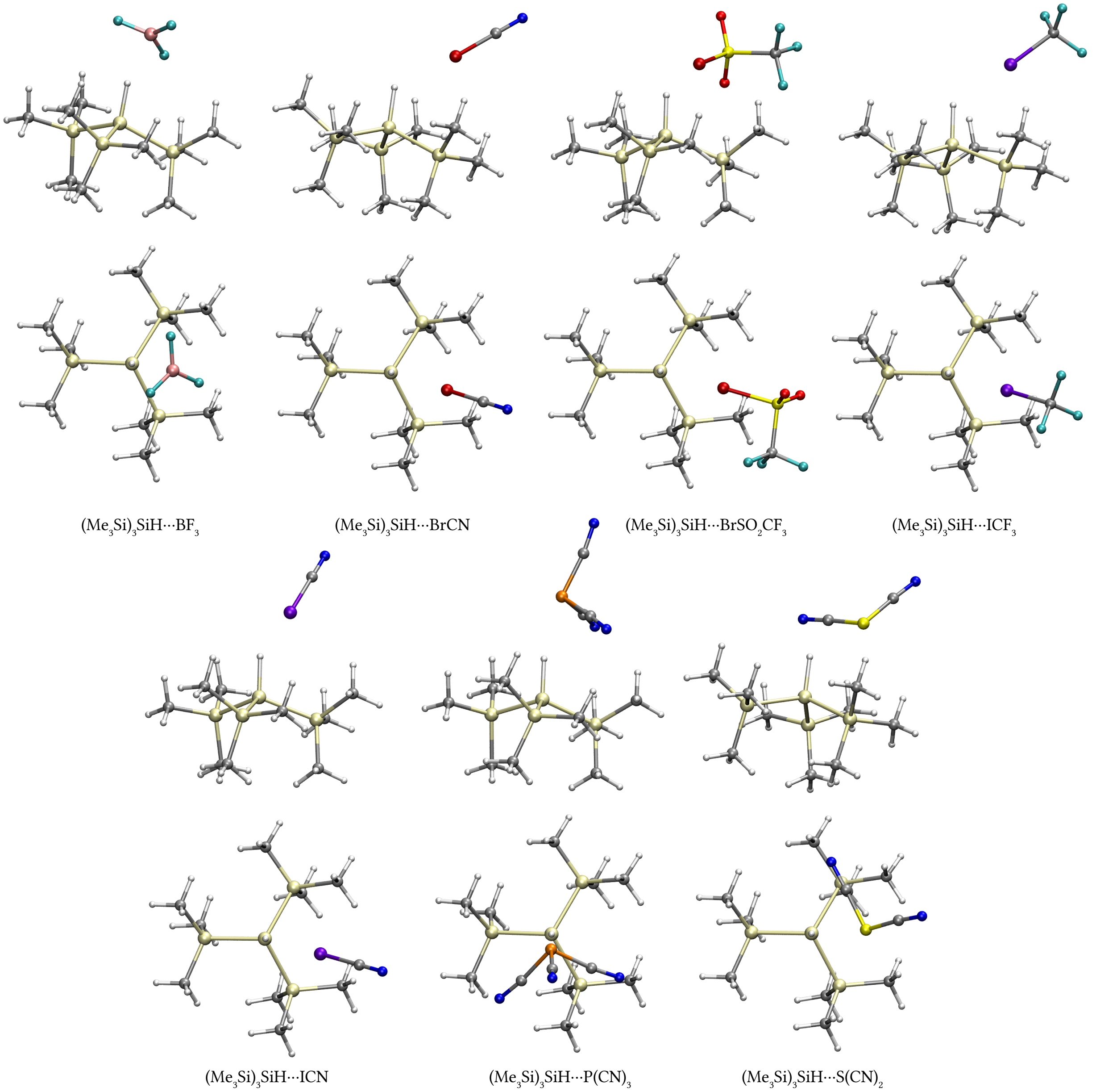

Figure S3: MP2/cc-pwCVTZ (cc-pwCVTZ-PP for B and I) optimized structures of red-shifting complexes of $(Me_3Si)_3SiH$ with BF_3, BrCN, $BrSO_2CF_3$, ICF_3, ICN, $P(CN)_3$, and $S(CN)_2$. [C: grey, Si: light yellow, H: white, B: pink, Br: dark red, F: cyan, I: purple, N: blue, O: red, P: orange, and S: yellow.]

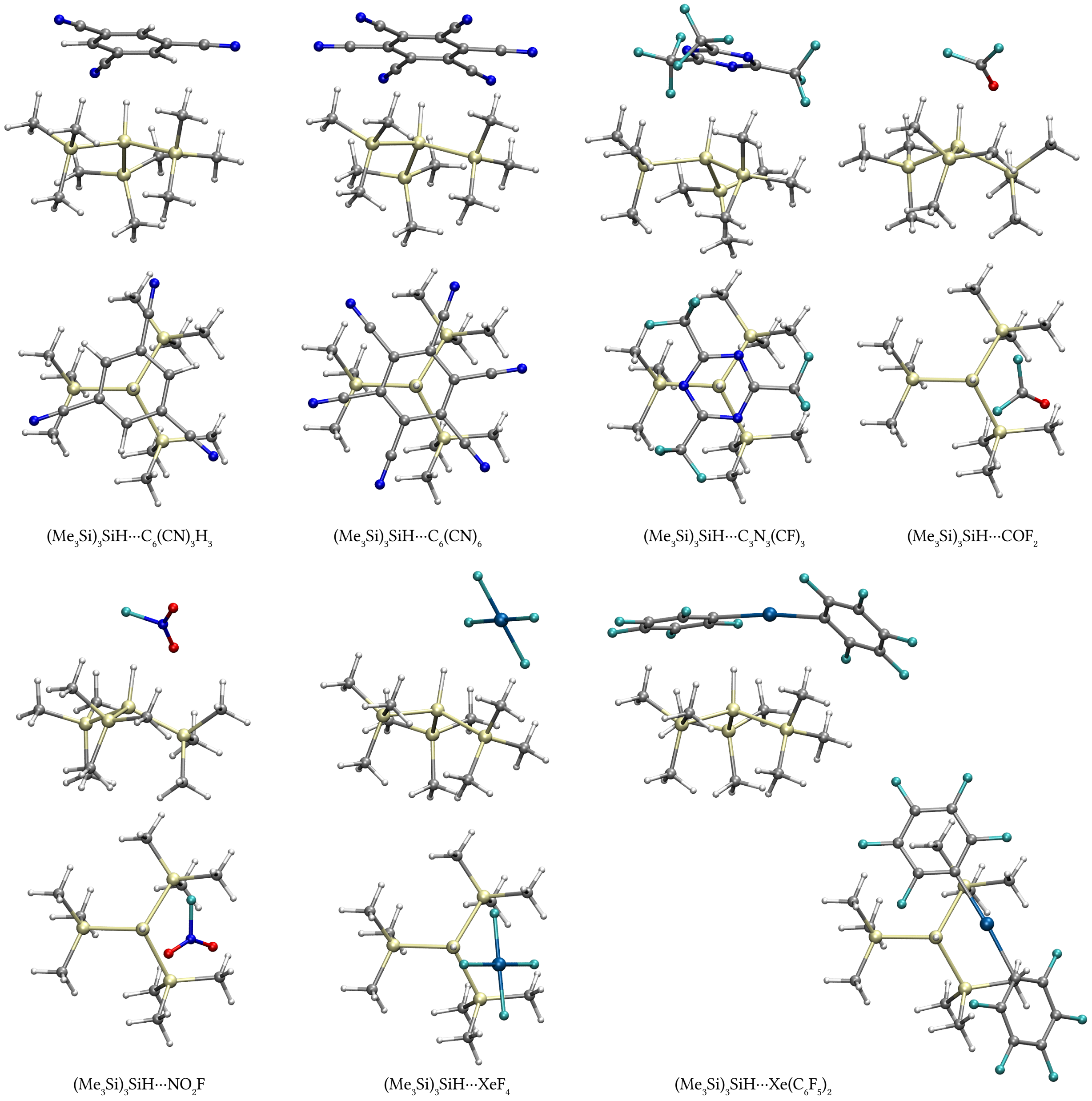

Figure S4: MP2/cc-pwCVTZ (cc-pwCVTZ-PP for Xe) optimized structures of blue-shifting complexes of $(Me_3Si)_3SiH$ with $C_6(CN)_3H_3$, $C_6(CN)_6$, $C_3N_3(CF_3)_3$, COF_2, NO_2F, XeF_4 and $Xe(C_6F_5)_2$. [C: grey, Si: light yellow, H: white, F: cyan, N: blue, O: red, and Xe: iceblue.]

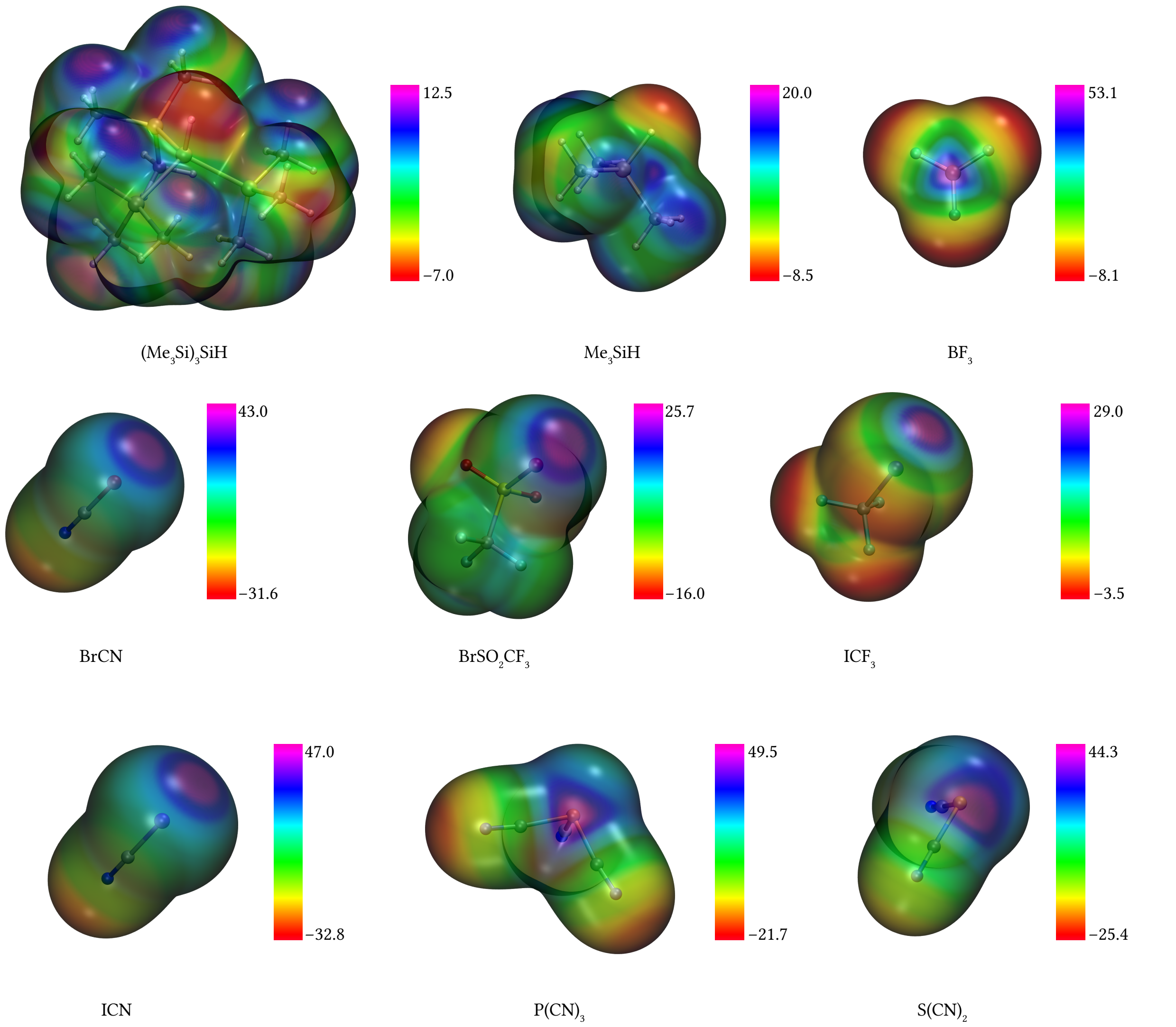

Figure S5: Molecular electrostatic potential for electron donors and σ-hole elctron acceptors calculated at MP2/cc-pwCVTZ (cc-pwCVTZ-PP for Br and I). Due to large range of $V_{\mathrm{s,min}}$ and $V_{\mathrm{s,max}}$, each molecule has its own color scale in kcal.mol^{-1}.

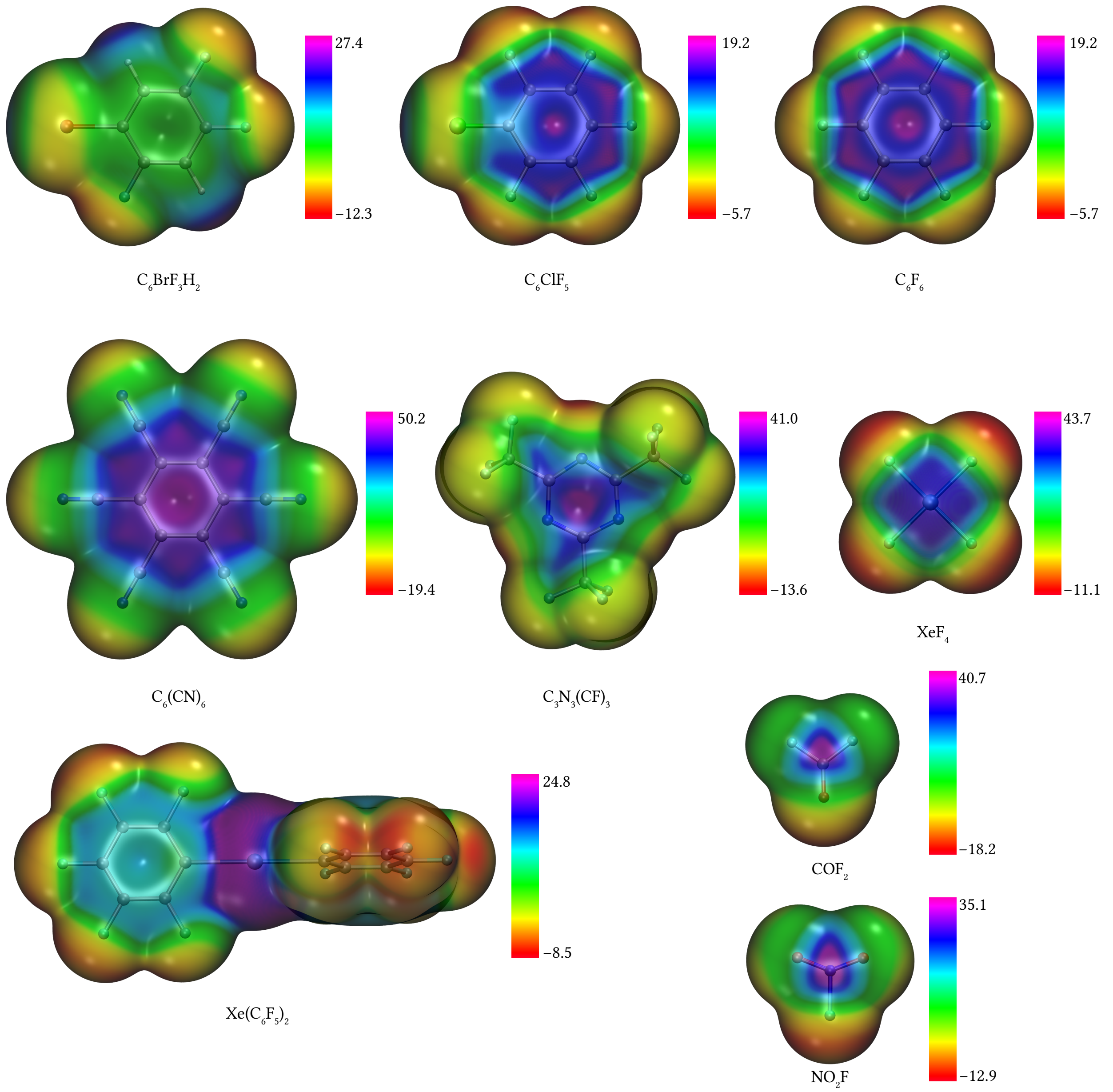

Figure S6: Molecular electrostatic potential for π-hole elctron acceptors calculated at MP2/cc-pwCVTZ (cc-pwCVTZ-PP for Xe). Due to large range of $V_{\mathrm{s,min}}$ and $V_{\mathrm{s,max}}$, each molecule has its own color scale in kcal.mol^{-1}.

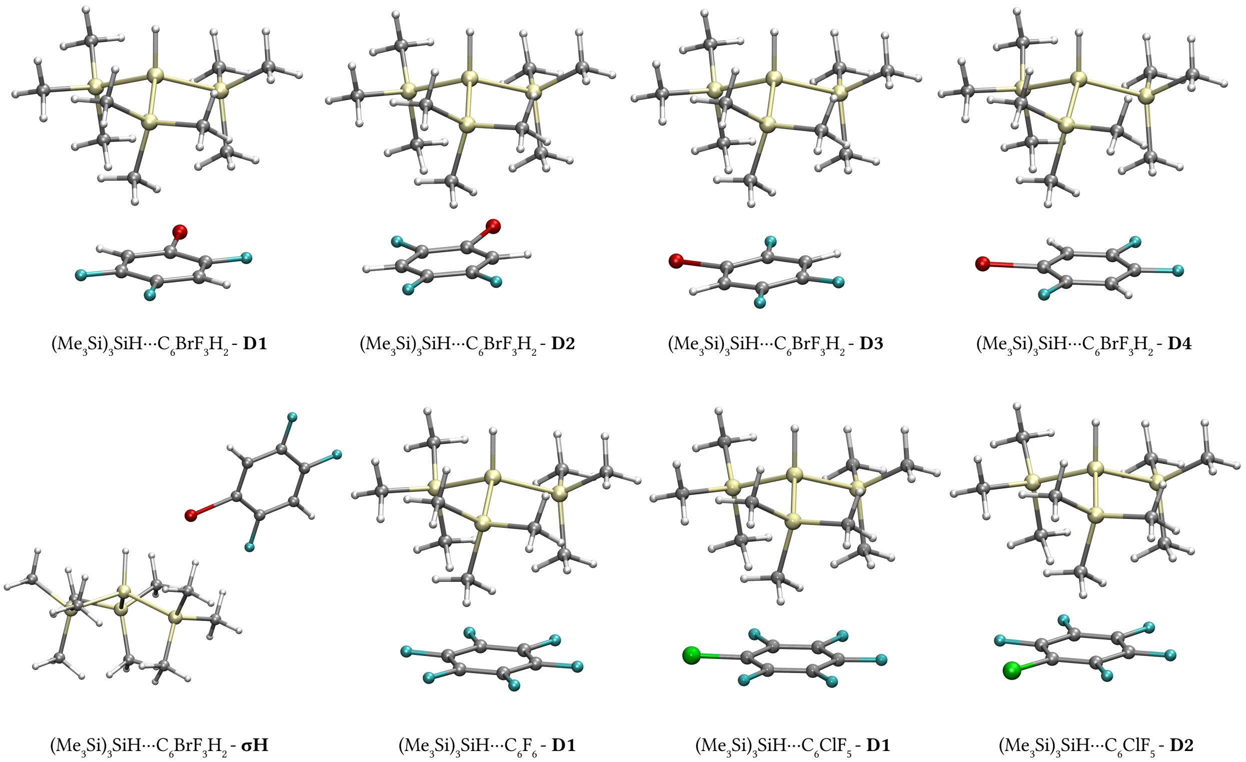

Figure S7: MP2/cc-pwCVTZ (cc-pwCVTZ-PP for Br) optimized structures of complexes of $(Me_3Si)_3SiH$ with $C_6BrF_3H_2$, C_6F_6 and C_6ClF_5 with other than Si-H$\cdots\pi$-hole contact. [C: grey, Si: light yellow, H: white, Br: dark red, Cl: green, and F: cyan.]

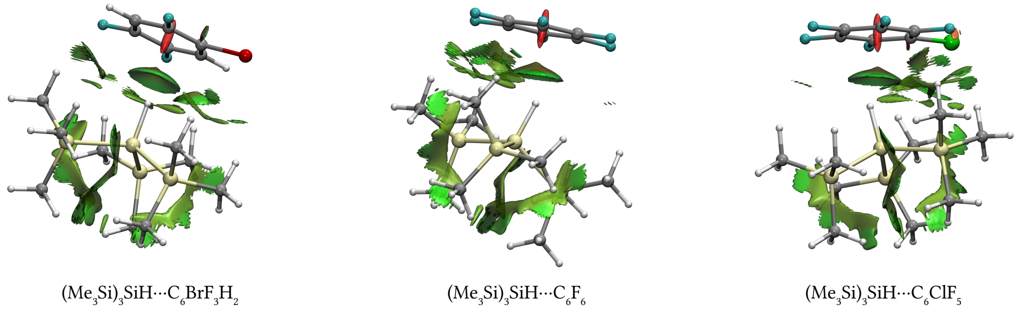

Figure S8: Gradient isosurfaces (s=0.5au) for complexes of $(Me_3Si)_3SiH$ with $C_6BrF_3H_2$, C_6F_6 and C_6ClF_5. The surfaces are colored on a blue-green-red scale according to values of $\text{sign}(\lambda_2)\rho$, ranging from -0.04 to 0.02au. Blue indicates strong attractive interactions, green indicates weak interactions, and red indicates strong non-bonded overlap. [C: grey, Si: light yellow, H: white, Br: dark red, Cl: green, and F: cyan.]

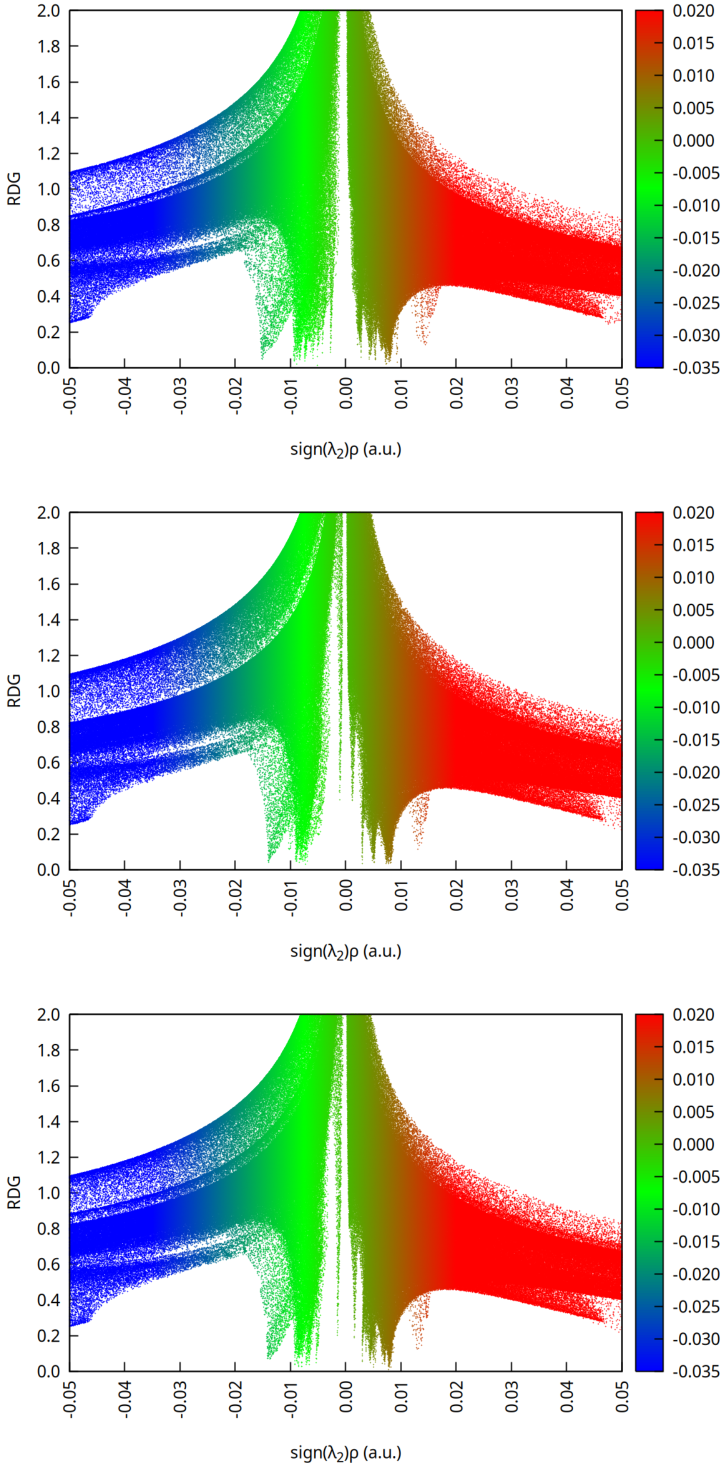

Figure S9: RDG scatter plots (right) of $Me_3Si)_3SiH$ with $C_6BrF_3H_2$ (top), C_6F_6 (middle) and C_6ClF_5 (bottom). Colour online: blue represents strong attractive interactions, green indicates van der Waals interactions and red indicates repulsive/steric interactions.

All optimized geometries are available at `https://github.com/mlamanec/BlueShiftingHydridicHydrogenBond`

Table S1: Benchmarking SAPT2+ against SAPT2+3 for the interaction energies of Me_3SiH complexes.[a]

Complex	SAPT level	$\Delta E_{\mathrm{Elstat}}$	ΔE_{Exch}	ΔE_{Ind}	ΔE_{Disp}	$\Delta E_{\mathrm{Total}}$
$Me_3SiH\cdots BF_3$ C_{3v} constrained	2+3/aug-cc-pwCVTZ[b]	−2.785	4.611	−1.355	−2.906	−2.434
	2+/aug-cc-pwCVTZ[b]	−2.832	4.611	−1.629	−2.711	−2.561
	2+/aug-cc-pwCVDZ[c]	−2.957	4.781	−1.610	−2.412	−2.198
$Me_3SiH\cdots BF_3$ bend	2+3/aug-cc-pwCVTZ[b]	−3.835	6.166	−1.289	−4.221	−3.178
	2+/aug-cc-pwCVTZ[b]	−3.912	6.166	−1.694	−4.011	−3.451
	2+/aug-cc-pwCVDZ[c]	−3.992	6.359	−1.652	−3.546	−2.831
$Me_3SiH\cdots ICF_3$ C_{3v} constrained	2+3/aug-cc-pwCVTZ[b]	−2.578	3.971	−0.433	−3.179	−2.219
	2+/aug-cc-pwCVTZ[b]	−2.562	3.971	−0.898	−3.014	−2.502
	2+/aug-cc-pwCVDZ[c]	−2.705	4.116	−0.962	−2.614	−2.165
$Me_3SiH\cdots ICF_3$ bend	2+3/aug-cc-pwCVTZ[b]	−2.774	5.339	−0.548	−4.781	−2.764
	2+/aug-cc-pwCVTZ[b]	−2.793	5.339	−1.219	−4.589	−3.261
	2+/aug-cc-pwCVDZ[c]	−2.911	5.554	−1.249	−3.973	−2.578
$Me_3SiH\cdots C_6F_6$ C_{3v} constrained	2+3/aug-cc-pwCVTZ[b]	−3.898	6.428	0.541	−5.926	−2.856
	2+/aug-cc-pwCVTZ[b]	−3.952	6.428	−0.086	−5.733	−3.342
	2+/aug-cc-pwCVDZ[c]	−4.096	6.608	−0.095	−5.348	−2.931
$Me_3SiH\cdots C_6F_6$ bend	2+3/aug-cc-pwCVTZ[b]	−3.930	8.614	0.234	−9.105	−4.186
	2+/aug-cc-pwCVTZ[b]	−4.086	8.614	−0.597	−8.874	−4.943
	2+/aug-cc-pwCVDZ[c]	−4.243	8.877	−0.593	−8.209	−4.168

[a] All values are in kcal.mol^{-1}

[b] aug-cc-pwCVTZ-PP for Br and I.

[c] aug-cc-pwCVDZ-PP for Br and I.

Table S2: Selected characteristics of hydridic hydrogen bond complexes evaluated at the PBE0-D4/def2-TZVPPD level.[a]

	ΔE^T	ΔG(20 K)	$\Delta\nu$(Si-H)	I_C/I_M[b]	Δr(Si-H)	$r(H\cdots X)$
$(Me_3Si)_3SiH\cdots BF_3$	−4.09	−3.15	−10.5	2.8	0.004	2.234
$(Me_3Si)_3SiH\cdots BrCN$	−3.96	−2.93	−37.1	2.8	0.006	2.663
$(Me_3Si)_3SiH\cdots BrSO_2CF_3$	−4.31	−3.40	−46.5	3.4	0.006	2.706
$(Me_3Si)_3SiH\cdots ICF_3$	−4.50	−3.66	−52.6	3.7	0.008	2.728
$(Me_3Si)_3SiH\cdots ICN$	−5.86	−4.82	−76.9	4.9	0.013	2.573
$(Me_3Si)_3SiH\cdots P(CN)_3$	−8.16	−7.02	−85.6	10.1	0.013	2.305
$(Me_3Si)_3SiH\cdots S(CN)_2$	−5.18	−4.19	−29.0	4.3	0.005	2.527
$(Me_3Si)_3SiH\cdots C_6F_6$	−5.49	−4.60	61.8	0.9	−0.005	2.458
$(Me_3Si)_3SiH\cdots C_6ClF_5$	−6.16	−5.41	65.0	0.9	−0.006	2.450
$(Me_3Si)_3SiH\cdots C_6BrF_3H_2$	−5.92	−5.62	55.0	0.7	−0.006	2.551
$(Me_3Si)_3SiH\cdots C_3(CN)_3H_3$	−7.23	−6.22	73.1	1.1	−0.007	2.430
$(Me_3Si)_3SiH\cdots C_6(CN)_6$	−10.26	−9.05	46.5	2.0	−0.001	2.399
$(Me_3Si)_3SiH\cdots C_3N_3(CF_3$	−8.11	−6.81	42.2	1.9	−0.002	2.478
$(Me_3Si)_3SiH\cdots COF_2$	−3.21	−2.21	−0.3	1.7	0.001	2.635
$(Me_3Si)_3SiH\cdots NO_2F$	−2.52	−1.57	4.6	1.3	0.000	2.753
$(Me_3Si)_3SiH\cdots XeF_4$	−4.59	−3.25	1.5	2.2	0.002	2.955
$(Me_3Si)_3SiH\cdots Xe(C_6F_5)_2$	−8.41	−7.30	31.8	1.4	−0.003	3.631

[a] ΔE^T and ΔG in kcal.mol^{-1}, $\Delta\nu$ in cm^{-1}, Δr and r in Å.
[b] Ratio between the intensity of the Si–H band in the complex (I_C) and that of the monomer (I_M).

Table S3: Selected characteristics of complexes of $(Me_3Si)_3SiH$ with $C_6BrF_3H_2$, C_6F_6 and C_6ClF_5 involving other than Si-H$\cdots$π-hole interaction calculated at MP2/cc-pwCVTZ (cc-pwCVTZ-PP for Br) level.[a]

Complex	ΔE^T	ΔG(20 K)	$\Delta\nu$(Si-H)	I_C/I_M[b]
$(Me_3Si)_3SiH\cdots C_6BrF_3H_2$ D1	−7.49	−6.44	2.0	1.1
$(Me_3Si)_3SiH\cdots C_6BrF_3H_2$ D2	−7.51	−6.52	2.4	1.1
$(Me_3Si)_3SiH\cdots C_6BrF_3H_2$ D3	−6.94	−5.93	2.3	1.1
$(Me_3Si)_3SiH\cdots C_6BrF_3H_2$ D4	−7.48	−6.48	2.8	1.1
$(Me_3Si)_3SiH\cdots C_6BrF_3H_2$ σH	−4.95	−4.08	−14.3	1.9
$(Me_3Si)_3SiH\cdots C_6F_6$ D1	−7.64	−6.44	4.6	1.1
$(Me_3Si)_3SiH\cdots C_6ClF_5$ D1	−8.16	−6.96	3.8	1.1
$(Me_3Si)_3SiH\cdots C_6ClF_5$ D2	−8.02	−6.92	4.4	1.1

[a] ΔE^T and ΔG in kcal.mol^{-1}, and ν in cm^{-1}.
[b] Ratio between the intensity of the Si–H band in the complex (I_C) and that of the monomer (I_M).

Table S4: Selected characteristics of complexes of $(Me_3Si)_3SiH$ with $C_6BrF_3H_2$, C_6F_6 and C_6ClF_5 involving other than Si-H$\cdots$π-hole interaction calculated at ωB97M-V/def2-TZVPPD level.[a]

Complex	ΔE^T	ΔG(20 K)	$\Delta\nu$(Si-H)	I_C/I_M[b]
$(Me_3Si)_3SiH\cdots C_6BrF_3H_2$ D1	−6.19	−5.83	7.0	1.1
$(Me_3Si)_3SiH\cdots C_6BrF_3H_2$ D2	−6.16	−5.60	7.1	1.1
$(Me_3Si)_3SiH\cdots C_6BrF_3H_2$ D3	−5.63	−5.06	7.8	1.1
$(Me_3Si)_3SiH\cdots C_6BrF_3H_2$ D4	−6.22	−5.52	9.9	1.1
$(Me_3Si)_3SiH\cdots C_6F_6$ D1	−5.95	−5.52	6.9	1.1
$(Me_3Si)_3SiH\cdots C_6ClF_5$ D1	−6.31	−5.99	7.8	1.1
$(Me_3Si)_3SiH\cdots C_6ClF_5$ D2	−6.27	−5.78	7.3	1.1

[a] ΔE^T and ΔG in kcal.mol^{-1}, and ν in cm^{-1}.
[b] Ratio between the intensity of the Si–H band in the complex (I_C) and that of the monomer (I_M).

Table S5: SAPT2+/aug-cc-pwCVDZ(aug-cc-pwCVTZ-PP for Br,I and Xe) energy decomposition of $(Me_3Si)_3SiH$ complexes.[a]

Complex	$\Delta E_{\mathrm{Elstat}}$	ΔE_{Exch}	ΔE_{Ind}	ΔE_{Disp}	$\Delta E_{\mathrm{Total}}$
$(Me_3Si)_3SiH\cdots BF_3$	−4.361	7.945	−2.129	−5.217	−3.762
$(Me_3Si)_3SiH\cdots BrCN$	−4.964	8.661	−1.347	−6.201	−3.852
$(Me_3Si)_3SiH\cdots BrSO_2CF_3$	−5.482	11.199	−1.181	−9.381	−4.845
$(Me_3Si)_3SiH\cdots ICF_3$	−4.745	10.230	−2.201	−7.527	−4.242
$(Me_3Si)_3SiH\cdots ICN$	−6.258	12.394	−3.366	−7.901	−5.131
$(Me_3Si)_3SiH\cdots PCl_3$	−3.111	8.576	−0.791	−8.344	−3.671
$(Me_3Si)_3SiH\cdots P(CN)_3$	−10.178	21.448	−6.317	−12.971	−8.018
$(Me_3Si)_3SiH\cdots S(CN)_2$	−6.981	14.299	−3.109	−9.608	−5.398
$(Me_3Si)_3SiH\cdots C_6F_6$	−7.385	14.502	−0.678	−12.717	−6.278
$(Me_3Si)_3SiH\cdots C_6ClF_5$	−7.543	15.286	−0.804	−13.856	−6.917
$(Me_3Si)_3SiH\cdots C_6BrF_3H_2$	−6.460	14.518	−0.877	−13.506	−6.325
$(Me_3Si)_3SiH\cdots C_3(CN)_3H_3$	−7.793	16.250	−1.086	−15.582	−8.211
$(Me_3Si)_3SiH\cdots C_6(CN)_6$	−10.096	21.530	−2.749	−20.952	−12.268
$(Me_3Si)_3SiH\cdots C_3N_3(CF_3$	−8.390	15.753	−1.537	−15.211	−9.385
$(Me_3Si)_3SiH\cdots COF_2$	−3.812	6.531	−0.986	−5.390	−3.656
$(Me_3Si)_3SiH\cdots NO_2F$	−3.874	6.697	−0.362	−6.249	−3.789
$(Me_3Si)_3SiH\cdots XeF_4$	−5.201	9.724	−1.757	−7.971	−5.206
$(Me_3Si)_3SiH\cdots Xe(C_6F_5)_2$	−8.810	21.920	−2.458	−21.995	−11.343

[a]All values are in $kcal.mol^{-1}$

Table S6: Selected characteristics of hydridic hydrogen bond complexes evaluated at the HF/cc-pwCVTZ (cc-pwCVTZ-PP for Br, I and Xe) and MP2/cc-pwCVTZ (cc-pwCVTZ-PP for Br, I and Xe) level.[a]

Complex	ΔE^{HF}	ΔE^{MP2}	$\Delta \nu^{\mathrm{HF}}$	$\Delta \nu^{\mathrm{MP2}}$	Δr^{HF}(Si-H)	Δr^{MP2}(Si-H)
$(Me_3Si)_3SiH\cdots BF_3$	−1.08	−4.09	−8.1	−0.5	0.002	0.002
$(Me_3Si)_3SiH\cdots BrCN$	−0.78	−4.47	−19.0	−29.2	0.004	0.005
$(Me_3Si)_3SiH\cdots BrSO_2CF_3$	−0.38	−5.48	−12.7	−29.3	0.002	0.004
$(Me_3Si)_3SiH\cdots ICF_3$	−0.50	−4.81	−12.3	−28.9	0.002	0.004
$(Me_3Si)_3SiH\cdots ICN$	−1.10	−5.86	−28.2	−48.2	0.005	0.008
$(Me_3Si)_3SiH\cdots P(CN)_3$	−1.23	−9.18	−27.6	−52.7	0.005	0.009
$(Me_3Si)_3SiH\cdots S(CN)_2$	−0.94	−6.32	−21.0	−21.1	0.004	0.004
$(Me_3Si)_3SiH\cdots C_6F_6$	−0.95	−9.34	0.5	78.1	0.001	−0.006
$(Me_3Si)_3SiH\cdots C_6ClF_5$	−0.88	−9.84	−6.8	72.6	0.002	−0.006
$(Me_3Si)_3SiH\cdots C_6BrF_3H_2$	−0.57	−7.81	−1.5	52.9	0.000	−0.004
$(Me_3Si)_3SiH\cdots C_3(CN)_3H_3$	−0.96	−9.98	−18.0	77.5	0.003	−0.006
$(Me_3Si)_3SiH\cdots C_6(CN)_6$	−1.39	−14.84	−55.7	57.9	0.009	−0.001
$(Me_3Si)_3SiH\cdots C_3N_3(CF_3$	−1.65	−11.91	−18.7	46.0	0.004	−0.001
$(Me_3Si)_3SiH\cdots COF_2$	−0.98	−4.20	−6.3	8.7	0.002	0.001
$(Me_3Si)_3SiH\cdots NO_2F$	−0.89	−4.76	−0.8	17.0	0.001	0.000
$(Me_3Si)_3SiH\cdots Xe$	−0.03	−2.23	0.4	8.0	0.000	−0.001
$(Me_3Si)_3SiH\cdots XeF_4$	−2.53	−7.18	−13.4	13.6	0.003	0.000
$(Me_3Si)_3SiH\cdots Xe(C_6F_5)_2$	−1.05	−13.49	−11.1	36.5	0.002	−0.003

[a]ΔE in kcal.mol^{-1},[a]$\Delta \nu$ in cm^{-1} and Δr in Å.

Table S7: Difference in occupancies in Si-H bonding orbital (σ(Si-H)), Si-H antibonding orbital (σ*(Si-H)), Si-Si bonding orbital (σ(Si-Si)) and Si-Si antibonding orbital (σ*(Si-Si)) between monomer $(Me_3Si)_3SiH$ and complexes of $(Me_3Si)_3SiH$ determined at ωB97M-V/def2-TZVPPD level of theory using NBO analysis.[a]

Complex	Δσ(Si-H)	Δσ*(Si-H)	Δσ(Si-Si)	Δσ(Si-Si)	Δσ(Si-Si)	Δσ*(Si-Si)	Δσ*(Si-Si)	Δσ*(Si-Si)
$(Me_3Si)_3SiH\cdots BF_3$	−0.009	0.004	0.001	0.000	0.000	0.001	0.002	0.002
$(Me_3Si)_3SiH\cdots BrCN$	−0.008	0.003	0.000	0.000	0.000	0.001	0.002	0.001
$(Me_3Si)_3SiH\cdots BrSO_2CF_3$	−0.006	0.002	0.000	0.000	−0.001	0.001	0.002	0.003
$(Me_3Si)_3SiH\cdots ICF_3$	−0.009	0.004	0.000	−0.001	0.000	0.002	0.003	0.001
$(Me_3Si)_3SiH\cdots ICN$	−0.017	0.006	0.001	−0.001	0.000	0.002	0.003	0.001
$(Me_3Si)_3SiH\cdots P(CN)_3$	−0.025	0.009	−0.001	−0.001	−0.001	0.003	0.002	0.001
$(Me_3Si)_3SiH\cdots S(CN)_2$	−0.010	0.004	0.000	0.000	0.000	0.002	0.002	0.001
$(Me_3Si)_3SiH\cdots C_6F_6$	−0.001	0.001	0.001	0.001	0.001	0.000	0.001	0.001
$(Me_3Si)_3SiH\cdots C_6ClF_5$	0.000	0.003	0.001	0.000	0.001	0.001	0.001	0.001
$(Me_3Si)_3SiH\cdots C_6BrF_3H_2$	0.000	0.003	0.001	0.001	0.001	0.001	0.001	0.001
$(Me_3Si)_3SiH\cdots C_3(CN)_3H_3$	−0.001	0.003	0.001	0.001	0.001	0.001	0.001	0.001
$(Me_3Si)_3SiH\cdots C_6(CN)_6$	−0.002	0.003	0.001	0.001	0.001	0.002	0.002	0.002
$(Me_3Si)_3SiH\cdots C_3N_3(CF_3$	0.000	0.003	0.001	0.001	0.000	0.002	0.002	0.001
$(Me_3Si)_3SiH\cdots COF_2$	−0.001	0.002	0.001	0.000	0.000	0.001	0.000	0.001
$(Me_3Si)_3SiH\cdots NO_2F$	0.000	0.002	0.001	0.000	0.000	0.001	0.001	0.001
$(Me_3Si)_3SiH\cdots XeF_4$	0.000	0.005	0.001	0.001	0.001	0.002	0.002	0.001
$(Me_3Si)_3SiH\cdots Xe(C_6F_5)_2$	−0.061	0.004	−0.063	−0.065	−0.064	0.002	0.002	0.002

[a]All values are in electrons.

Table S8: Second-order perturbation energies from NBO analysis (E_2) for charge transfer from electron donor to electron acceptor, evaluated at ωB97M-V/def2-TZVPPD. Only values larger than 1 kcal.mol^{-1} are shown. No charge-transfer interactions greater than 1 kcal.mol^{-1} were found in the electron-acceptor → electron-donor direction. For complexes not displayed, no values larger than 1 kcal.mol^{-1} were detected.[a]

Complex	Direction of Charge Transfer	E_2
$(Me_3Si)_3SiH\cdots BF_3$	σ(Si-H) → σ*(B-F1)	1.00
	σ(Si-H) → σ*(B-F2)	1.05
$(Me_3Si)_3SiH\cdots BrCN$	σ(Si-H) → σ*(Br-C)	2.12
$(Me_3Si)_3SiH\cdots BrSO_2CF_3$	σ(Si-H) → σ*(Br-S)	1.40
$(Me_3Si)_3SiH\cdots ICF_3$	σ(Si-H) → σ*(I-C)	2.54
$(Me_3Si)_3SiH\cdots ICN$	σ(Si-H) → σ*(I-C)	4.27
$(Me_3Si)_3SiH\cdots P(CN)_3$	σ(Si-H) → σ*(P-C)	3.24
$(Me_3Si)_3SiH\cdots S(CN)_2$	σ(Si-H) → σ*(S-C)	1.73

[a] E_2 in kcal.mol^{-1}.

Table S9: Selected characteristics of CCl_3H hydrogen bond complexes evaluated at the ωB97M-V/def2-TZVPPD level with full optimization (FULL), optimization on polarized surface (POL) and optimization on freezed surface (FRZ) using adiabatic ALMO-EDA method. [a]

Complex	FULL $\Delta\nu$	FULL I_C/I_M [b]	FULL Δr	POL $\Delta\nu$	POL I_C/I_M [b]	POL Δr	FRZ $\Delta\nu$	FRZ I_C/I_M [b]	FRZ Δr
$CCl_3H\cdots MeOH$	−0.8	88.6	0.0010	41.6	38.3	−0.0014	48.9	2.1	−0.0019
$CCl_3H\cdots H_2O$	−8.7	13.1	0.0010	11.9	3.9	−0.0003	13.1	1.2	−0.0004
$CCl_3H\cdots NH_3$	−64.6	190.3	0.0044	36.5	52.0	−0.0011	54.1	2.1	−0.0022
$CCl_3H\cdots PH_3$	−3.9	10.2	0.0006	13.8	3.0	−0.0004	13.6	1.2	−0.0005
$CCl_3H\cdots C_6H_6$	27.1	37.3	−0.0003	55.7	21.4	−0.0020	48.8	2.4	−0.0023
$CCl_3H\cdots C_6FH_5$	32.1	36.2	−0.0010	57.7	16.8	−0.0023	47.9	2.3	−0.0024
$CCl_3H\cdots H_2S$	13.6	38.9	−0.0003	37.1	18.7	−0.0018	38.9	1.8	−0.0019

[a] $\Delta\nu$ in cm^{-1}, and Δr in Å.

[b] Ratio between the intensity of the Si–H band in the complex (I_C) and that of the monomer (I_M).

Table S10: Selected characteristics of Me_3SiH hydridic hydrogen bond complexes evaluated at the ωB97M-V/def2-TZVPPD level with full optimization (FULL), optimization on polarized surface (POL) and optimization on freezed surface (FRZ) using adiabatic ALMO-EDA method. [a]

Complex	FULL			POL			FRZ		
	$\Delta\nu$	I_C/I_M[b]	Δr	$\Delta\nu$	I_C/I_M[b]	Δr	$\Delta\nu$	I_C/I_M[b]	Δr
$Me_3SiH\cdots BF_3$	−39.9	1.7	0.0093	−24.4	1.4	0.0057	−21.8	1.0	0.0046
$Me_3SiH\cdots BrCN$	−44.3	1.8	0.0083	−25.6	1.4	0.0048	−18.7	1.0	0.0038
$Me_3SiH\cdots C_6F_6$	−4.3	1.1	0.0031	−4.3	1.1	0.0032	−5.5	1.0	0.0027
$Me_3SiH\cdots C_6H_3(CN)_3$	−5.9	1.1	0.0040	−7.4	1.1	0.0039	−7.0	0.9	0.0036
$Me_3SiH\cdots COF_2$	−28.5	1.4	0.0062	−22.5	1.3	0.0050	−21.1	1.0	0.0044
$Me_3SiH\cdots ICF_3$	−33.6	2.1	0.0078	−14.0	1.5	0.0039	−8.5	1.0	0.0029
$Me_3SiH\cdots ICN$	−69.7	2.4	0.0140	−38.9	1.6	0.0081	−31.3	1.0	0.0059
$Me_3SiH\cdots NO_2F$	−16.1	1.2	0.0041	−15.9	1.2	0.0038	−15.2	1.0	0.0034
$Me_3SiH\cdots P(CN)_3$	−85.3	3.4	0.0155	−35.3	1.9	0.0089	−30.8	1.0	0.0066
$Me_3SiH\cdots S(CN)_2$	−56.5	2.0	0.0103	−33.8	1.5	0.0069	−27.3	1.0	0.0055
$Me_3SiH\cdots XeF_4$	−29.7	1.6	0.0068	−21.3	1.5	0.0055	−20.1	1.0	0.0044

[a] $\Delta\nu$ in cm^{-1}, and Δr in Å.

[b] Ratio between the intensity of the Si–H band in the complex (I_C) and that of the monomer (I_M).

Table S11: Selected stretching frequencies and corresponding band intensities of complexes with σ-hole electron acceptors calculated at ωB97M-V/def2-TZVPPD level.[a]

Complex	Vibration type	ν_{MONOMER}	ν_{COMPLEX}	$\Delta\nu$	I_{MONOMER}	I_{COMPLEX}	$I_{\mathrm{C}}/I_{\mathrm{M}}$[b]
$(Me_3Si)_3SiH\cdots BrCN$	Br-C	601.4	594.1	−7.3	0.5	2.5	5.0
$(Me_3Si)_3SiH\cdots BrSO_2CF_3$	Br-S	294.0	293.9	0.1	0.6	0.2	0.3
$(Me_3Si)_3SiH\cdots ICF_3$	I-C	297.1	295.6	−1.5	0.3	2.6	8.7
$(Me_3Si)_3SiH\cdots ICN$	I-C	514.0	501.8	−12.2	2.0	18.8	9.4
$(Me_3Si)_3SiH\cdots P(CN)_3$	P-C antisymm.	662.6	649.1	−13.5	70.2	110.5	1.6
	P-C antisymm.	663.1	660.4	−2.7	70.4	45.6	0.6
$(Me_3Si)_3SiH\cdots S(CN)_2$	S-C antisymm.	705.3	705.4	0.1	3.2	4.5	1.4

[a] ν in cm^{-1}.

[b] Ratio between the intensity of the Si–H band in the complex (I_{C}) and that of the monomer (I_{M}).

Table S12: Si-H stretching frequency [ν(Si-H)] and its respective intensity [I(Si-H)] evaluated at the MP2/cc-pwCVTZ (cc-pwCVTZ-PP for Br, I and Xe), ωB97M-V/def2-TZVPPD and PBE0-D4/def2-TZVPPD level.[a]

	MP2		ωB97M-V		PBE0	
	ν(Si-H)	I(Si-H)	ν(Si-H)	I(Si-H)	ν(Si-H)	I(Si-H)
$(Me_3Si)_3SiH$ monomer	2202.29	83.38	2161.77	109.06	2136.45	88.64
$(Me_3Si)_3SiH\cdots BF_3$	2201.78	187.18	2150.29	247.62	2125.96	248.25
$(Me_3Si)_3SiH\cdots BrCN$	2173.09	205.99	2145.84	258.70	2099.33	248.90
$(Me_3Si)_3SiH\cdots BrSO_2CF_3$	2172.96	195.87	2130.95	235.21	2089.92	305.32
$(Me_3Si)_3SiH\cdots ICF_3$	2173.42	216.43	2138.48	286.72	2083.89	330.86
$(Me_3Si)_3SiH\cdots ICN$	2154.13	290.47	2133.35	400.03	2059.53	437.28
$(Me_3Si)_3SiH\cdots P(CN)_3$	2149.59	513.16	2118.12	651.55	2050.82	895.84
$(Me_3Si)_3SiH\cdots S(CN)_2$	2181.22	234.93	2152.86	328.37	2107.44	379.51
$(Me_3Si)_3SiH\cdots C_6F_6$ **II**	2280.36	59.98	2203.87	120.01	2198.29	79.79
$(Me_3Si)_3SiH\cdots C_6ClF_5$ **IIIa**	2274.89	58.81	2207.20	118.52	2200.03	78.66
$(Me_3Si)_3SiH\cdots C_6ClF_5$ **IIIb**	2272.91	59.59	2222.84	110.75	2201.42	77.72
$(Me_3Si)_3SiH\cdots C_6BrF_3H_2$ **Ia**	2255.15	48.65	2227.46	90.54	2200.98	63.16
$(Me_3Si)_3SiH\cdots C_6BrF_3H_2$ **Ib**	2260.32	46.39	2232.43	85.68	2191.47	66.45
$(Me_3Si)_3SiH\cdots C_6BrF_3H_2$ **Ic**	2274.07	42.05	2225.58	89.80	2204.58	61.19
$(Me_3Si)_3SiH\cdots C_6BrF_3H_2$ **Id**	2270.43	45.69	2231.30	85.95	2198.12	64.30
$(Me_3Si)_3SiH\cdots C_3(CN)_3H_3$	2279.78	73.02	2236.20	125.23	2209.57	96.10
$(Me_3Si)_3SiH\cdots C_6(CN)_6$	2260.24	138.52	2210.93	208.63	2182.96	177.65
$(Me_3Si)_3SiH\cdots C_3N_3(CF_3$	2248.25	151.98	2216.40	193.05	2178.66	166.61
$(Me_3Si)_3SiH\cdots COF_2$	2211.03	129.87	2168.43	175.04	2136.12	153.92
$(Me_3Si)_3SiH\cdots NO_2F$	2219.30	98.17	2173.24	137.03	2141.02	116.70
$(Me_3Si)_3SiH\cdots XeF_4$	2215.85	170.01	2162.87	213.63	2137.93	193.78
$(Me_3Si)_3SiH\cdots Xe(C_6F_5)_2$	2238.83	104.13	2190.89	141.58	2168.24	126.36

[a] ν in cm^{-1}, I in $km.mol^{-1}$.

Table S13: Si-H stretching frequency [ν(Si-H)] and its respective intensity [I(Si-H)] evaluated at the ωB97M-V/def2-TZVPPD level with full optimization (FULL), optimization on polarized surface (POL) and optimization on freezed surface (FRZ) using adiabatic ALMO-EDA method.[a]

	FULL		POL		FRZ	
	ν(Si-H)	I(Si-H)	ν(Si-H)	I(Si-H)	ν(Si-H)	I(Si-H)
CCl_3H monomer	3189.25	1.21				
$CCl_3H \cdots MeOH$	3188.46	107.13	3230.85	46.26	3238.19	2.57
$CCl_3H \cdots H_2O$	3180.57	15.86	3201.13	4.68	3202.38	1.48
$CCl_3H \cdots NH_3$	3124.63	230.10	3225.73	62.87	3243.33	2.55
$CCl_3H \cdots PH_3$	3185.38	12.30	3203.01	3.62	3202.86	1.44
$CCl_3H \cdots C_6H_6$	3216.31	45.13	3244.97	25.83	3238.03	2.87
$CCl_3H \cdots C_6FH_5$	3221.39	43.79	3246.97	20.35	3237.14	2.82
$CCl_3H \cdots H_2S$	3202.89	47.06	3226.30	22.62	3228.15	2.15
Me_3SiH monomer	2207.16	183.22				
$Me_3SiH \cdots BF_3$	2167.24	315.77	2182.81	248.82	2185.37	182.00
$Me_3SiH \cdots BrCN$	2162.90	327.28	2181.60	262.45	2188.49	181.08
$Me_3SiH \cdots C_6F_6$	2202.86	196.45	2202.85	203.41	2201.67	176.57
$Me_3SiH \cdots C_6H_3(CN)_3$	2201.25	207.01	2199.79	207.39	2200.20	172.45
$Me_3SiH \cdots COF_2$	2178.62	249.48	2184.62	230.53	2186.02	188.28
$Me_3SiH \cdots ICF_3$	2173.60	378.76	2193.14	272.67	2198.67	180.35
$Me_3SiH \cdots ICN$	2137.43	441.97	2168.22	298.86	2175.87	182.84
$Me_3SiH \cdots NO_2F$	2191.10	223.03	2191.28	216.45	2191.92	181.83
$Me_3SiH \cdots P(CN)_3$	2121.82	626.32	2171.86	347.40	2176.36	176.38
$Me_3SiH \cdots S(CN)_2$	2150.69	364.26	2173.34	269.18	2179.89	180.18
$Me_3SiH \cdots XeF_4$	2177.51	297.30	2185.83	268.52	2187.04	179.60
$(Me_3Si)_3SiH$ monomer	2161.77	109.06				
$(Me_3Si)_3SiH \cdots BF_3$	2150.29	247.62	2161.96	179.42	2157.85	102.04
$(Me_3Si)_3SiH \cdots BrCN$	2145.84	258.70	2159.29	182.29	2158.29	104.37
$(Me_3Si)_3SiH \cdots BrSO_2CF_3$	2130.95	235.21	2156.15	159.64	2160.31	106.81
$(Me_3Si)_3SiH \cdots ICF_3$	2138.48	286.72	2153.24	192.18	2163.17	106.16
$(Me_3Si)_3SiH \cdots ICN$	2133.35	400.03	2158.99	245.87	2153.21	104.48
$(Me_3Si)_3SiH \cdots P(CN)_3$	2118.12	651.55	2172.95	315.13	2169.66	96.70
$(Me_3Si)_3SiH \cdots S(CN)_2$	2152.86	328.37	2171.13	221.85	2164.76	101.11
$(Me_3Si)_3SiH \cdots C_6F_6$	2203.87	120.01	2201.33	136.44	2196.86	90.98
$(Me_3Si)_3SiH \cdots C_6ClF_5$	2207.20	118.52	2209.40	134.11	2197.78	89.91
$(Me_3Si)_3SiH \cdots COF_2$	2168.43	175.04	2173.16	155.89	2170.25	102.63
$(Me_3Si)_3SiH \cdots NO_2F$	2173.24	137.03	2178.47	139.09	2174.29	100.59
$(Me_3Si)_3SiH \cdots XeF_4$	2162.87	213.63	2165.80	195.47	2163.40	101.35
$(Me_3Si)_3SiH$ monomer [b]	2162.93	105.62				
$(Me_3Si)_3SiH \cdots C_6BrF_3H_2$ [b]	2225.45	82.26	2223.91	105.82	2218.30	84.22

[a] ν in cm^{-1}, I in $km.mol^{-1}$.
[b] in def2-TZVPP basis set.
